# Xuanqing Hefa formula relieves sepsis-triggered acute lung injury by targeting the NLRP3/Caspase-1 pyroptosis mechanism

**DOI:** 10.3389/fimmu.2025.1709586

**Published:** 2026-02-03

**Authors:** Can Lei, Chunxia Zhao, Xucheng Li, Kangli Wang, Tianfen Cen, Qingquan Liu, Xiaolong Xu, Jun Zhang

**Affiliations:** 1 Hubei University of Chinese Medicine, Wuhan, China; 2 Wuhan Hospital of Traditional Chinese Medicine, Wuhan, China; 3 National Medical Hospital of Hubei University of Traditional Chinese Medicine, Wuhan, China; 4 The Second People's Hospital of Jingzhou, Jingzhou, China; 5 Tongren Hospital of Traditional Chinese Medicine, Tongren, China; 6 Beijing Hospital of Traditional Chinese Medicine, Capital Medical University, Beijing, China; 7 Beijing Institute of Chinese Medicine, Beijing, China; 8 Beijing Key Laboratory of Basic Research with Traditional Chinese Medicine on Infectious Diseases, Beijing, China; 9 Laboratory for Clinical Medicine, Capital Medical University, Beijing, China

**Keywords:** acute lung injury, NLRP3 inflammasome, pyroptosis, sepsis, Xuanqing Hefa formula

## Abstract

**Introduction:**

Acute lung injury (ALI), frequently triggered by sepsis, is characterized by severe respiratory distress and high mortality, particularly in critically ill or elderly individuals. Xuanqing Hefa formula (XQHF) has been clinically applied to alleviate ALI by reducing pulmonary inflammation; however, its underlying mechanisms remain poorly understood. This study aimed to evaluate the therapeutic effects of XQHF on sepsis-induced ALI using both *in vitro* and *in vivo* models, and to explore its potential mechanisms of action.

**Materials and methods:**

Multiple active constituents of XQHF were identified using UPLC-MS/MS. To investigate the therapeutic potential of XQHF against sepsis-induced lung injury, a cecal ligation and puncture (CLP) model was established in mice, while an *in vitro* pyroptosis model was developed using lipopolysaccharide (LPS) and Nigericin (Nig) stimulation in Immortalized bone marrow-derived macrophages (iBMDMs). The protective effects of XQHF were evaluated through seven-day survival analysis, histopathological examination of lung tissue, pulmonary wet-to-dry weight ratio assessment (W/D), and quantification of total protein in bronchoalveolar lavage fluid (BALF). In addition, inflammatory responses and cellular injury were assessed via ELISA, oxidative stress assays, lactate dehydrogenase (LDH) release, and immunofluorescence staining. The anti-pyroptotic effects of XQHF were further elucidated using western blotting and flow cytometry.

**Results:**

The results demonstrated that XQHF significantly preserved lung tissue architecture by attenuating both inflammatory responses and oxidative stress, thereby improving the survival rate of septic mice. At the molecular level, XQHF inhibited the expression of key pyroptosis-related proteins. Similar protective effects were observed in the LPS + Nigericin-induced pyroptosis model in iBMDMs, where XQHF also reduced apoptosis. Confocal microscopy further confirmed that XQHF markedly decreased the expression of pro-inflammatory cytokines, including IL-1β and IL-18.

**Conclusions:**

Our results suggest that the therapeutic effects of XQHF may be attributed to the synergistic actions of its multiple active constituents, which collectively suppress the inflammatory cascade and attenuate pyroptosis, thereby alleviating lung injury. Furthermore, both *in vivo* and *in vitro* experiments demonstrated that XQHF modulates sepsis-induced acute lung injury, at least in part, through the inhibition of the NLRP3/Caspase-1-dependent pyroptosis signaling pathway.

## 1 Introduction

Sepsis is a life-threatening medical emergency marked by catastrophic organ dysfunction caused by an overactive immune response to infection. This devastating condition poses a significant global health challenge, accounting for an estimated 48.9 million cases and nearly 11 million deaths annually worldwide (1). Research shows that sepsis most often targets the lungs, with pulmonary infections occurring in 40–60% of cases, sepsis-induced ALI often advances to ARDS, significantly increasing mortality risk (2). In various etiologies of ALI/ARDS, lung injury consistently correlates with macrophage pyroptosis (3, 4). Overactivated macrophages undergo pyroptosis during sepsis, triggering excessive inflammatory responses and oxidative radical production. While this aids pathogen clearance, it simultaneously damages pulmonary microvascular endothelial cells (PMVECs) (5), ultimately leading to pathological alterations such as enhanced pulmonary inflammatory cell infiltration, increased cellular necrosis, and exacerbated tissue edema/exudation. The cornerstone of managing ALI/ARDS remains supportive care, which prioritizes lung-protective ventilation strategies and careful fluid management. These approaches are often supplemented with adjunct therapies like glucocorticoids, surfactant administration, and Extracorporeal Membrane Oxygenation (ECMO) when necessary. Despite these interventions, significant hurdles remain—patients frequently face grim outcomes, lasting cognitive deficits, and diminished life quality post-recovery (6). Therefore, developing targeted pharmacological interventions for ALI is urgently needed to mitigate tissue damage, modulate immune responses, and control inflammation.

The NLRP3 inflammasome—a cellular signaling powerhouse responsible for spurring on inflammatory agents—is made up of NLRP3, ASC (a protein that looks a bit like a speck), and Caspase-1. This complex is like the immune system’s ace in the hole, crucial for combating infections. Researchers have tied it to the roots of several widespread diseases that aren’t contagious, such as lung damage from sepsis, encephalopathy caused by sepsis, diabetes, and atherosclerosis (3, 7–9). NLRP3 inflammasome assembly triggers Caspase-1 activation, initiating IL-1β and IL-18 release, gasdermin D cleavage, and ultimately pyroptosis. NLRP3 inflammasome activation has two stages: priming and activation. It has been shown that NLRP3 inflammasome activation consists of two phases: priming and activation. Priming, usually involving macrophages and TLR ligands like LPS, primes the NF-κB pathway, increasing NLRP3 and IL-1β. Activation then occurs with stimuli like ATP and nigericin (10). Research showed that GSDMD-deficient mice suppressed IL-1β and LDH release in LPS + Nig-induced pyroptosis of bone marrow-derived macrophages (11). Traditional Chinese medicine (TCM) therapies have been demonstrated to mitigate the inflammatory response by reducing the activation of NLRP3 inflammasome (12, 13). Consequently, suppressing NLRP3 inflammasome activity and preventing pyroptosis may offer a hopeful therapeutic approach for addressing ALI. To assess the effectiveness of the experimental treatment protocol, this research employed caffeic acid (CA) and disulfiram (DSF) as positive controls in both *in vivo* and *in vitro* studies. CA, a potent phenolic acid, exhibits antioxidant and anti-inflammatory properties. Studies show it lowers IL-1β and TNF-α levels in LPS-induced septic mice while providing *in vitro* antioxidant effects. Additionally, CA inhibits pyroptosis by suppressing GSDMD upregulation, mitigating LPS-induced sepsis (14). DSF was demonstrated to block the development of membrane holes from cleaved GSDMD, obstructing cytokine release and cellular focalization, and reducing LPS-induced septic mortality in mice (15, 16). While both medications show promise, they aren’t without limitations. Caffeic acid’s potential for treating ALI remains understudied, with a notable lack of robust clinical trials and insufficient evidence to confirm its therapeutic effectiveness. Studies have shown that disulfiram can cause the accumulation of acetaldehyde, leading to flushing and other aversive reactions (17). Therefore, we need to explore new effective methods for treating ALI.

Xuanqing Hefa Formula (XHQF) is an in-hospital preparation that has been used for many years at Wuhan Hospital of Traditional Chinese Medicine. Derived from Xiaochaihu Decoction and Yinqiao Powder, XHQF is modified for the treatment of ALI and contains traditional Chinese medicinal ingredients such as *Bupleuri Radix* (Chaihu), *Scutellariae Radix* (Huangqin), and *Isatidis Folium* (Daqingye). It is currently a registered hospital-prepared formulation at this institution. The theoretical foundation of XHQF originates from *Shang Han Lun* by Zhongjing Zhang, and *Wenbing Tiaobian* by Tang Wu. According to TCM theory, sepsis arises from the interaction between exogenous pathogens and endogenous toxic factors, with the core pathogenesis being “excess pathogens and deficient healthy qi, leading to stasis-toxin obstructing collaterals.” XHQF follows the therapeutic principles of “dispelling pathogens, clearing heat, and harmonizing the exterior and interior.” Clinical observations have demonstrated that XHQF, when combined with conventional supportive therapy, mitigates inflammation and modulates immune responses, exhibiting significant efficacy in ameliorating sepsis-induced lung injury and COVID-19-related pulmonary complications. Nonetheless, foundational research regarding the processes that underlie the impact of XHQF on ALI is insufficient.

**Figure 1:**
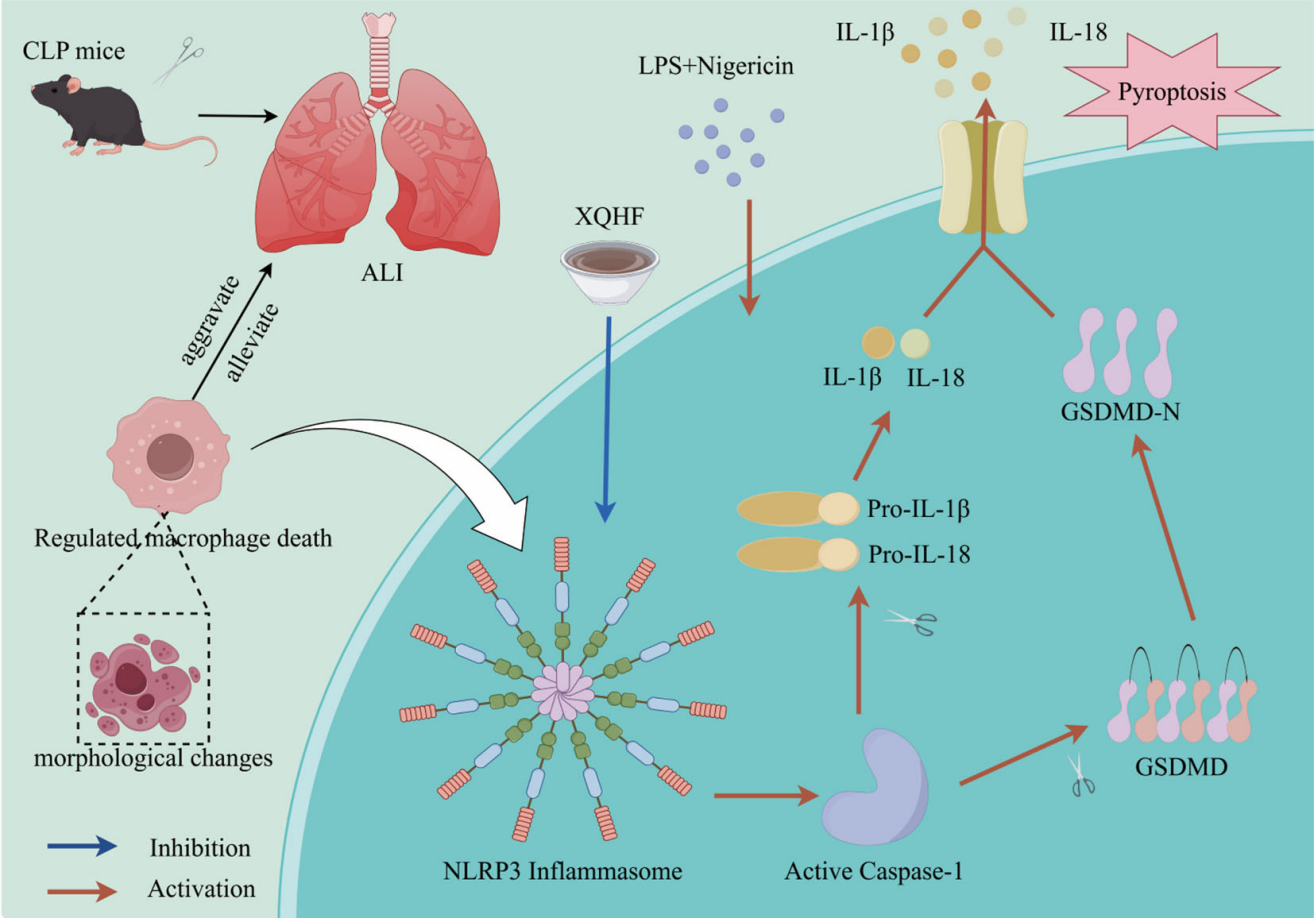
Schematic of XQHF’s inhibition of pyroptosis and inflammation via NLRP3/Caspase-1 pathway modulation.

By referring to the research on sepsis conducted by Hsu et al. (3, 18, 19), we also adopted the CLP mouse model and the LPS + Nig-induced classical pathway macrophage pyroptosis model to investigate the mechanism by which XQHF regulates NLRP3 inflammasome activation. We assessed the therapeutic efficacy of XQHF on ALI to offer novel insights and perspectives for sepsis treatment. **Figure 1** delineates the intricate workflow diagram that constitutes the foundational framework of the present investigation.

## 2 Materials and methods

### 2.1 Preparation of XQHF

The XQHF formula consists of the following Chinese medicinal materials (medicinal Latin name, original plant Latin name, dose): Bupleuri Radix (Radix Bupleuri, *Bupleurum chinense* DC. or *Bupleurum scorzonerifolium* Willd.) 15 g; Scutellariae Radix (Radix Scutellariae, *Scutellaria baicalensis* Georgi) 15 g; Isatidis Folium (Folium Isatidis, *Isatis indigotica Fortune*) 15 g; Scrophulariae Radix (Radix Scrophulariae, *Scrophularia ningpoensis* Hemsl.) 20 g; Pinelliae Rhizoma (Rhizoma Pinelliae, *Pinellia ternata (Thunb.)* Breit., processed as Rhizoma Pinelliae Preparata) 10 g; Arctii Fructus (Fructus Arctii, *Arctium lappa* L.) 10 g; Forsythiae Fructus (Fructus Forsythiae, *Forsythia suspensa* (Thunb.) Vahl) 10 g; Taraxaci Herba (Herba Taraxaci, *Taraxacum mongolicum* Hand.-Mazz.) 20 g; Cyrtomii Rhizoma (Rhizoma Cyrtomii, *Cyrtomium fortunei* J. Sm.) 10 g; Nepetae Herba (Herba Nepetae, *Nepeta cataria* L.) 10 g; Glycyrrhizae Radix et Rhizoma (Radix et Rhizoma Glycyrrhizae, *Glycyrrhiza uralensis* Fisch.) 10 g; Isatidis Radix (Radix Isatidis, *Isatis indigotica* Fortune) 10 g; Poria (Poria, *Wolfiporia cocos* (Schwein.) Ryvarden & Gilb.) 15 g; Armeniacae Semen Amarum (Semen Armeniacae Amarum, *Prunus armeniaca* L. var. *ansu* Maxim.) 12 g. All doses are presented in grams (g). The herbal materials, sourced from Wuhan Hospital of Traditional Chinese Medicine, weighed exactly 182 grams. These ingredients were steeped in 500 mL of purified water for 30 minutes before undergoing a 40-minute boiling process. The initial decoction was then separated, and the remaining herbs were boiled again with fresh purified water for an additional 20 minutes. The extracts, combined, were strained via a two-ply gauze and then condensed to 73 mL, yielding a 2.5 g/mL concentration of the herbal infusion. This high-potency preparation served as the maximum dosage (XQHF) for murine subjects, which could be diluted with purified water to create intermediate (1.25 g/mL) and low (0.625 g/mL) concentration variants. All prepared solutions were refrigerated at 4°C.

### 2.2 Experimental animals and modeling

For this study, we used male C57BL/6J mice from Beijing Huafukang Biotechnology. These mice, a robust *in vitro* model, were selected for their ease of use and consistent genetics. Health-screened at procurement, they ensure high-quality, unbiased research. The animals were eight weeks old at the start of experimentation, with body weights ranging between 20–22 g. The experimental protocols were meticulously designed and rigorously implemented in full compliance with internationally recognized standards for laboratory animal welfare. Prior to commencement, the study underwent comprehensive ethical review and received formal approval from the independent Animal Ethics Committee at the Beijing Institute of Traditional Chinese Medicine (Approval Reference: BJTCM-M-2024-09-03), ensuring alignment with the “3Rs” principles (Replacement, Reduction, and Refinement). Every step of the process followed established ethical guidelines to ensure proper treatment of research animals. To develop the sepsis model, we conducted CLP using 1% sodium pentobarbital for anesthesia. A small midline incision, approximately 1–2 cm in length, was made to expose the cecum. The exposed portion was then ligated at its central point, effectively obstructing about 50% of the total cecal length. The sterile needle punctured the cecum, discharging a minimal quantity of chyme to ascertain the procedure’s completion. The cecum was then repositioned and the abdominal wall closed using absorbable and non-absorbable sutures/staples. This method reduces postoperative risks and facilitates precise intestinal manipulation. Postoperatively, each mouse received 2 mL of sterile saline (0.9%) subcutaneously for rehydration. Sham-operated controls in experimental research undergo laparotomy without ligations or punctures to mimic the intervention without treatment effects. This maintains study integrity, allowing for direct comparison and accurate assessment of the intervention’s efficacy and complications.

### 2.3 Treatment protocol for XQHF

Mice were acclimated for seven days under controlled conditions before the experiment, with standardized food, clear water, and normal sleep to reduce stress. After modification, mice were assigned randomly to six groups, each consisting of 15 equivalent subjects, ensuring equal sample sizes and reducing potential bias for accurate comparisons. The sham group underwent mock surgery without any medication, while the CLP group underwent the surgical procedure without receiving XQHF therapy. Three dosage tiers of XQHF were administered post-CLP surgery: low (12.5 g/kg body weight), medium (25 g/kg), and high (50 g/kg). A separate group received 30 mg/kg of CA following the same surgical protocol. Six hours post-operation, the XQHF-treated mice were given 0.4 mL of their respective doses orally, whereas the CA group received an equivalent volume via intraperitoneal injection. The sham and CLP groups received 0.4 mL oral saline. Treatments were repeated every 24 hours over a seven-day survival observation period. The experimental design was then replicated, with tissue samples collected 24 hours after the final administration.

### 2.4 Sample collection

24 hours following the establishment of the mouse model, tissue and fluid samples were harvested. Prior to specimen collection, all necessary equipment was prepared, including sterilized ophthalmic surgical tools, cryovials, disposable syringes, pre-sterilized centrifuge tubes, medical gauze, saline solution, liquid nitrogen, and 4% tissue fixative. Mice were anesthetized with 1% pentobarbital sodium for deep sedation, allowing for a minimally invasive thoracotomy to collect blood from the right ventricle. Blood samples were allowed to clot for 2 hours, subjected to centrifugation for serum isolation, and subsequently frozen at -80°C. Following blood collection, a tracheal cannulation was performed using a needle secured with 4–0 silk sutures. Bronchoscopy-assisted lung lavage employed 1 mL of chilled, sterile saline; the acquired BALF was then preserved at -20°C for later examination. Following bronchial lavage, the harvested lung tissue specimens were immediately cryopreserved by rapid immersion in liquid nitrogen to halt cellular degradation and preserve biomolecular integrity. The cryogenically stabilized samples were then transferred to a ultra-low temperature freezer maintained at -80°C for long-term archival storage. For histological examination, non-lavaged lung tissue was gently rinsed with saline, submerged in 4% tissue fixative, and refrigerated at 4°C until further processing.

### 2.5 Cell culture and treatment

Professor Liu’s research team at the Beijing Institute of Chinese Medicine generously provided the iBMDMs. IBMDMs were incubated in DMEM (GIBCO, USA), supplemented with 10% fetal bovine serum and 1% penicillin/streptomycin, and maintained at 37°C in a 5% CO_2_ environment within a humidified setup. The research involved a few distinct groups: a baseline control group, a group exposed to LPS alone at a concentration of 1 μg/mL, one group treated with LPS plus Nig, and three additional groups that were treated with varying percentages of serum rich in XQHF (namely, 5%, 10%, and 20%). Additionally, an LPS + Nig + DSF group (10 μM, Targetmol) was included. To model NLRP3 inflammasome activation, LPS (L2630, Sigma) and nigericin (N849347, Macklin) were employed. The adherent iBMDM cells were first pretreated with XQHF and DSF, respectively, for 2 h. After removal of the pretreatments, the cells were stimulated with LPS (1 μg/mL) for 4 h. Finally, Nig (20 μM) was added to the culture. This secondary treatment was carried out over an additional two-hour incubation period to induce further inflammatory signaling. The sequential application of these stimulants—first LPS to prime the inflammasome pathway, followed by nigericin to potentiate caspase-1 activation—represents a well-established experimental model for studying NLRP3 inflammasome activation *in vitro*. Notably, DSF was co-incubated with both LPS and nigericin throughout the treatment period.

### 2.6 Preparation of XQHF-containing serum

To minimize discrepancies, a total of 30 male Sprague-Dawley rats (Approval Reference: BJTCM-R-2024-09-02) were haphazardly segregated into the control and XQHF treatment groups, evenly split, with every effort made to maintain their comparable weights and ages. Over seven consecutive days, the control animals received ultra-pure water via oral gavage, while the experimental group was administered XQHF at a dosage of 50 g/kg daily. After the final treatment, the rats were subjected to a standardized anesthesia protocol, including intraperitoneal injection of a 1% sodium pentobarbital solution. Upon successful induction of anesthesia, blood was drawn from the rats via the abdominal aorta through an abdominal incision. The blood collected from this major arterial catheter was immediately transferred into sterile tubes. The blood samples underwent centrifugation at a speed of 3,000 rpm for a full 10 minutes, all the while chilling at a balmy 4°C, with the goal of extracting the serum. Serum was heat-inactivated at 56 °C for 30 min in a water bath. Subsequently, it was sterilized by passage through a 0.22 µm filter, aliquoted, and stored at -80 °C until use.

### 2.7 UPLC-MS/MS of XQHF

The XQHF components were uploaded into specialized metabolomics analysis software for processing. A series of computational procedures—including baseline adjustment, compound identification, data integration, retention time normalization, and peak alignment—were executed to generate a comprehensive data matrix. This matrix contained detailed analytical parameters such as retention times, mass-to-charge ratios, and peak intensity values. Following this preprocessing stage, the software’s search algorithms were employed to detect and characterize the distinctive spectral peaks. Then, the LC-MS analysis of XQHF, conducted on a UHPLC-Q Exactive platform, yielded mass spectrometry data that was cross-referenced against an in-house database of traditional Chinese medicine metabolites. The mass accuracy threshold was maintained at under 10 ppm for reliable compound identification. Further confirmation of metabolite structures was achieved through evaluation of MS/MS spectral matching scores.

### 2.8 Hematoxylin-eosin staining

Slice the lung tissue into thin slivers, approximately as fine as a piece of paper (around 4–5 micrometers). Place the slivers in an oven to remove the paraffin. Next, immerse the slices in a series of ethanol solutions, starting with 100%, then 95%, and finishing with 70%, followed by a dip in pure H_2_O. Once this process is complete, proceed with the HE staining. After the staining is finished, rinse the slices briefly to remove excess eosin. Then, allow the slices to dry completely. Soak them in xylene to clear the tissue, and they will be ready for examination under the microscope to observe their tissue structure.

### 2.9 Lung tissue wet/dry ratio

After an intensive 24-hour course of modeling, the study subjects, which were mice, were humanely euthanized. Subsequent to this procedure, their lungs were meticulously excised and carefully weighed in their natural, unprocessed state. Following this, the lungs were subjected to a drying process, which was conducted within an oven set to a precise temperature of 75°C for a duration of 48 hours. The weight of the lung tissue after drying was recorded, and this information was then used in conjunction with the original wet weight to compute the wet-to-dry weight ratio. This statistical metric serves as a crucial indicator for evaluating the extent of pulmonary edema.

### 2.10 Measurement of inflammatory factors and total protein in BALF

BALF, serum, and cell culture supernatants were centrifuged at 3000 rpm for 15 minutes at 4°C to isolate debris. Clarified samples were analyzed the levels of inflammatory factors (IL-18, IL-1β, IL-6, and TNF-α) via ELISA (Laizee Biotech kits), following manufacturer protocols with sample dilution and duplicate measurements. The absorbance of the sample was precisely quantified at a wavelength of 450 nm using a high-throughput microplate spectrophotometer. This specific wavelength was selected due to its optimal sensitivity for detecting the chromogenic reaction product under investigation. The microplate reader, equipped with a monochromator-based optical system, ensured accurate and reproducible measurements across all sample replicates. BALF protein content was quantified using a BCA assay (Thermo Fisher), with BSA standards. Samples’s absorbance were measured at 562 nm. Protein normalization ensured accurate cytokine comparisons. Controls validated assay precision.

### 2.11 Cell viability

IBMDMs in the logarithmic growth phase were carefully seeded into sterile 96-well plates at a density of 1 × 10^5^ cells per well. Following incubation for 24 h under standard culture conditions (37°C, 5% CO_2_), the cells were treated with different concentrations of blank serum, XQHF-containing serum, LPS + Nig alone, LPS + Nig combined with blank serum, or LPS + Nig combined with XQHF-containing serum, respectively. For cell viability assessment, 10 µl of the CCK-8 solution (CK04, Dojindo Laboratories, Japan) was added to each well. After an additional incubation period (typically 2–4 h; please specify the exact duration according to the manufacturer’s protocol), the absorbance at 450 nm was measured using a microplate spectrophotometer.

### 2.12 LDH assay

LDH serves as a reliable biomarker for assessing cell membrane integrity, providing valuable insights into cellular mortality or injury while also serving as an indirect measure of pyroptosis activation. During the experiment, logarithmic grown iBMDM cells were carefully seeded into 12-well plates at an optimal density of 5 × 10^5^ cells/well. Following successful induction of the standard pyroptosis model, the culture medium was harvested and subjected to centrifugation 3000 rpm for 15 minutes at 4°C to isolate debris. The resulting supernatant was carefully transferred to 1.5 mL microcentrifuge tubes. Then, a commercial LDH assay kit (CK12, Dojindo Laboratories, Japan) was used to detect LDH release.

### 2.13 Western blot

For optimal protein extraction, lung tissues and iBMDM cells were thoroughly lysed using RIPA buffer (APPLYGEN, Beijing, China) supplemented with protease inhibitors (APPLYGEN, Beijing, China), followed by vigorous homogenization. The lung tissues were then centrifuged to collect the supernatant for subsequent analysis. Protein concentrations were quantified via BCA assay, after which equal amounts were resolved on SDS-PAGE gels. Electrophoretically separated proteins were then transferred to PVDF membranes (Millipore) using standard protocols. Membranes were blocked and probed overnight at 4°C with the following primary antibodies diluted in blocking buffer: NLRP3 (1:1000, P60622R3, Abmart, Shanghai), ASC (1:10,000, 10500-1-AP, Proteintech, Wuhan), Caspase-1 (1:1000, 24,232, Cell Signaling Technology, USA), GSDMD (1:1000, Ab219800, Abcam, UK), IL-18 (1:2000, 10663-1-AP, Proteintech, Wuhan), and IL-1β (1:1000, Ab234437, Abcam, UK). Following primary incubation, membranes were washed three times with TBST and then incubated for 40 minutes at room temperature with an HRP-conjugated secondary antibody (goat anti-rabbit IgG, 1:10,000, RGAR001, Proteintech, Wuhan). After additional TBST washes, protein bands were detected using enhanced chemiluminescence (Millipore, USA). β-actin (1:20,000, 60008-1-Ig, Proteintech, Wuhan) was used as a loading control to ensure equal protein loading across samples.

### 2.14 Measurement of oxidative factors

To prepare mouse lung tissue samples, first homogenize the tissue using ice-cold RIPA lysis buffer (APPLYGEN, Beijing, China). Spin the homogenate at 12,000 revolutions per minute for 10 minutes at 4°C, then carefully pour the clarified fluid into a new EP tube for subsequent analysis. For enzymatic and oxidative stress marker analysis, aliquot the tissue supernatant into 96-well plates and follow the manufacturer’s protocols to measure NO, MPO, SOD, CAT, ROS, and MDA levels using a microplate reader. Standard curves should be generated for accurate quantification. The SOD and MPO detection kits were sourced from Beijing Solarbio Science & Technology Co., Ltd., while the NO, CAT, ROS, and MDA detection kits were obtained from Beyotime Institute of Biotechnology.

### 2.15 Flow cytometry (FCM)

Following exposure to different experimental conditions, the iBMDMs were carefully detached with trypsin, collected by centrifugation, and rinsed twice with phosphate-buffered saline (PBS). The cell pellets were subsequently reconstituted in 200 μL of Binding Buffer—a process repeated in triplicate to ensure thorough preparation. Following this, we added 5 microliters of propidium iodide (PI) staining solution, which had a concentration of 100 micrograms per milliliter, along with 10 microliters of FITC Annexin V staining solution. The mixture was then incubated for a quarter of an hour at ambient temperature, all while keeping it in the dark. An additional 300 μL of Binding Buffer was added, and the mixture was left to incubate for an hour. Apoptosis levels were ultimately assessed using flow cytometry with a FACS system.

### 2.16 Immunofluorescence staining

Immunofluorescence analysis verified the expression of inflammatory indicators IL-18 and IL-1β in the corresponding groups of iBMDMs. The fixed cell slides were briefly rinsed for 10 minutes in 4% paraformaldehyde, followed by a 5-minute permeabilization with 0.2% Triton X-100. Post that, we let the samples sit with goat serum for a solid hour at room temp to block any non-specific binding. Then, we kept them chilled at 4°C overnight, using IL-18 (10663-1-AP, Proteintech, Wuhan) and IL-1β (Ab234437, Abcam, UK) primary antibodies at a 1:500 dilution, all while keeping the light out. The next day, we added FITC-labeled secondary antibodies against rabbit and mouse, diluted 1:800, and let them incubate for an hour at room temp. Cellular fluorescence was visualized using laser confocal microscopy, with quantitative assessment performed through the Image Pro Plus 6.0 software. Three random microscopic fields were evaluated to determine average fluorescence intensity in order to analyze accurately.

### 2.17 Statistical analysis

The study analyzed data rigorously, presenting results as mean ± standard deviation (SD) to reflect central tendency and variability. Analyses used GraphPad Prism 8, a reliable statistical tool. One-way ANOVA is utilized to assess normally distributed data with homogeneity of variances, effectively identifying statistical significance. “ns” denotes non-significant differences (likely due to chance), while “*p* < 0.05” indicates statistically significant results, suggesting meaningful effects. This method ensures robust, reliable conclusions, advancing knowledge in the field.

## 3 Results

### 3.1 UPLC-MS/MS analysis of XQHF

**Figure 2:**
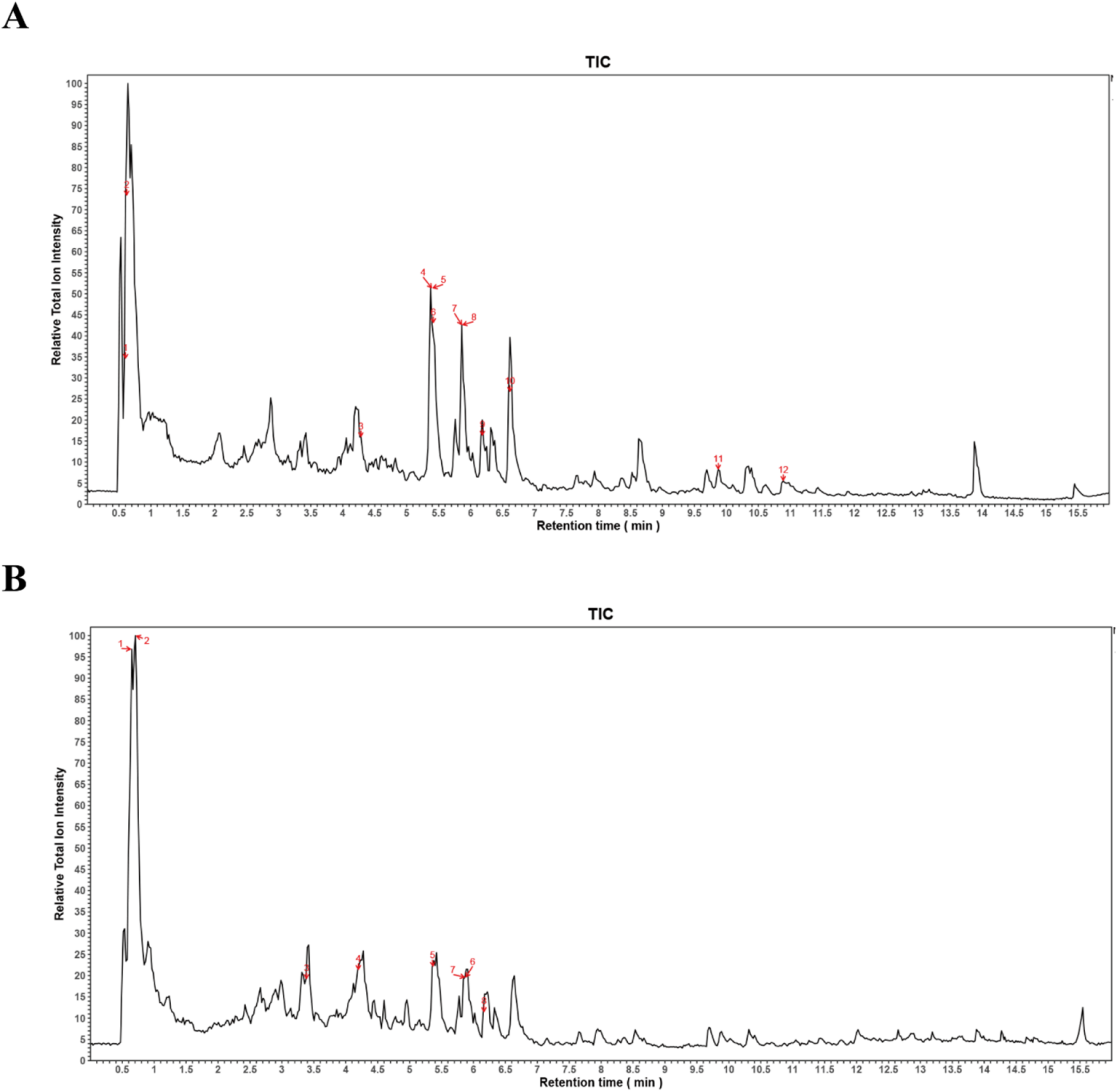
XQHF was identified in both negative and positive ion modes within the total ion chromatogram via UPLC-MS/MS analysis. **(A)** Negative ion mode in sample-XQHF. **(B)** Positive ion mode in sample-XQHF.

Identified key chemical constituents in the extracts and evaluated XQHF purity using UPLC-MS/MS. **Figure 2** displays the total ion chromatogram (TIC). Numerous compounds were detected in XQHF, including different categories, such as organic acids, alkaloids, coumarins, flavonoids, monoterpenes, cyclic enol ether terpenoids, and other components. The top 20 compounds with XQHF content are shown in **Table 1**.

**Table 1:** Top 20 compounds detected in XQHF using UPLC-MS/MS analysis.

Compound.	tR	Molecular formula	Name	Origin
1	5.39535	C_21_H_18_O_11_	Glychionide A	*Scutellaria baicalensis* Georgi
2	6.615133333	C_22_H_20_O_11_	Wogonoside	*Scutellaria baicalensis* Georgi
3	4.1973	C_29_H_36_O_15_	Forsythoside I	*Forsythia suspensa* (Thunb.) Vahl
4	6.18325	C_22_H_20_O_11_	Oroxyloside	*Scutellaria baicalensis* Georgi
5	5.36805	C_21_H_18_O_11_	Baicalin	*Scutellaria baicalensis* Georgi
6	0.602033333	C_6_H_14_N_4_O_2_	L-arginine	*Isatis indigotica Fortune*
7	0.706566667	C_6_H_8_O_7_	Citric acid	*Taraxacum mongolicum* Hand.-Mazz.
8	5.877116667	C_21_H_18_O_11_	Apigenin 7-O-beta-D-glucuronide	*Scutellaria baicalensis* Georgi
9	5.39535	C_15_H_10_O_5_	Aloe emodin	*Scutellaria baicalensis* Georgi
10	3.384633333	C_20_H_27_NO_11_	Amygdalin	*Prunus armeniaca* L. var. *ansu* Maxim.
11	6.165083333	C_22_H_20_O_11_	Oroxylin A-7-O-beta-D-glucuronide	*Scutellaria baicalensis* Georgi
12	0.619833333	C_5_H_9_NO_2_	L-proline	*Scrophularia ningpoensis* Hemsl.
13	5.86145	C_21_H_24_O_6_	Arctigenin	*Arctium lappa* L.
14	9.872416667	C_16_H_12_O_5_	Acacetin	*Scutellaria baicalensis* Georgi
15	5.416916667	C_15_H_10_O_6_	Scutellarein	*Scutellaria baicalensis* Georgi
16	0.650366667	C_4_H_6_O_5_	D-(+)-Malic acid	*Scrophularia ningpoensis* Hemsl.
17	5.877116667	C_18_H_22_O_11_S	Paederoside	*Isatis indigotica Fortune*
18	10.88906667	C_16_H_32_O_2_	14-Methylpentadecanoic acid	*Prunus armeniaca* L. var. *ansu* Maxim.
19	5.86145	C_21_H_24_O_6_	Phillygenin	*Forsythia suspensa* (Thunb.) Vahl
20	4.283716667	C_15_H_12_O_4_	Isoliquiritigenin	*Glycyrrhiza uralensis* Fisch

### 3.2 XQHF augmented survival chances and ameliorated lung injury in CLP-induced septic mice

**Figure 3:**
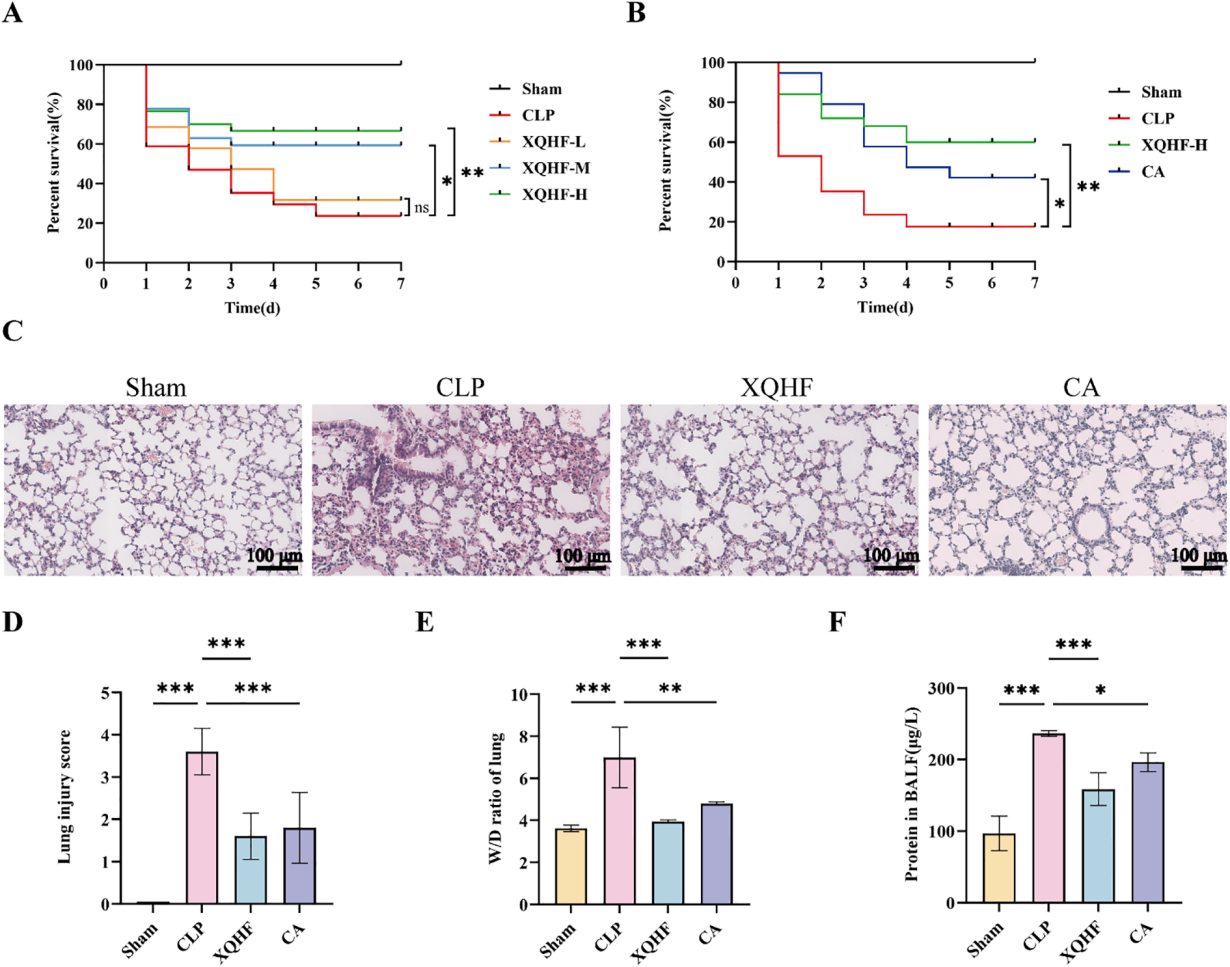
XQHF enhances survival chances and mitigates lung damage in CLP-induced septic mice. **(A, B)** Seven-day survival curve (n = 15, each group). **(C)** H&E staining results of lung tissues in each group (n = 5). **(D)** Lung injury pathology score was assessed (n = 5). **(E)** Lung W/D ratios were calculated in the indicated groups (n = 5). **(F)** BALF protein levels were measured with a BCA assay kit (n = 5). Values are expressed as mean ± SD, ns, no significance, *p < 0.05, **p < 0.01, ***p < 0.001.

Monitoring the seven-day survival outcomes in septic mice allowed us to evaluate the impact of XQHF on mortality rates. The data revealed that while the septic mice exhibited expected mortality patterns, the low-dose XQHF treatment (XQHF-L) did not show any statistically significant improvement in survival outcomes. Nonetheless, mice in the XQHF-M and XQHF-H cohorts exhibited a notably greater 7-day survival rate, and XQHF-H was more effective (**Figure 3A**, p < 0.01). For the follow-up experiments, XQHF-H was selected to evaluate how XQHF and CA treatments impacted the survival rates of CLP mice. The findings revealed that while CA enhanced the 7-day survival rate compared to untreated CLP-induced septic mice, its effectiveness fell short of XQHF’s superior results (**Figure 3B**, p < 0.01).

H&E staining was used to assess CLP-induced lung damage in mice, revealing key markers of ALI/ARDS such as edema, alveolar flooding, neutrophil infiltration, and fibrosis. Compared to the sham group, CLP mice demonstrated notable pathological alterations, including pronounced exudation, bleeding, infiltration of inflammatory cells, damage to alveolar walls, and widespread lung injury (**Figure 3C**). Remarkably, treatment with XQHF or CA significantly reduced lung tissue damage in sepsis mice due to CLP (**Figure 3C**), with a pronounced enhancement observed in the XQHF therapy. Regarding lung damage scores, mice in the XQHF and CA groups had significantly lower lung damage scores than CLP mice (**Figure 3D**, p < 0.001). W/D and protein concentration in BALF are important parameters for evaluating lung edema and microvascular permeability. We found that CLP-induced septic mice significantly increased W/D and protein content in BALF (**Figures 3E, F**), but a significant decrease after treatment with XQHF (50 g/kg). CA had a similar effect but was not as therapeutic as XQHF. These results suggest that XQHF has a significant protective effect.

### 3.3 XQHF abated the inflammatory response and oxidative stress *in vivo*

**Figure 4:**
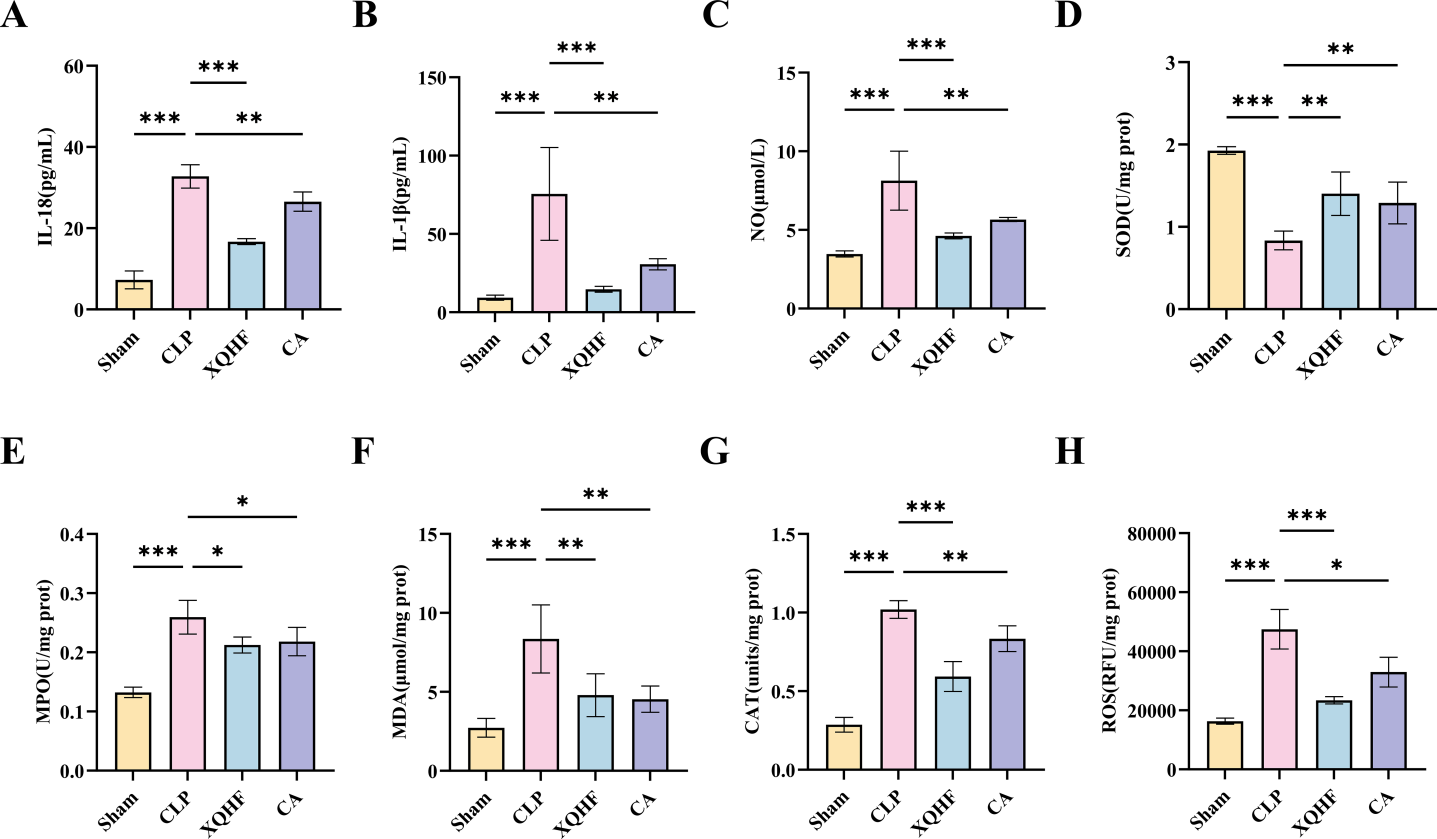
XQHF abated the inflammation and oxidative stress in CLP-induced sepsis mice. **(A, B)** Activity levels of IL-18 and IL-1β in lung tissue were determined by ELISA (n = 5). **(C)** Levels of NO in lung tissue was determined by NO assay kit (n = 5). **(D–H)** Oxidative stress in mice lung tissue was assessed by analyzing SOD, MDA, MPO, CAT, and ROS levels across the specified groups (n = 5). Values are expressed as mean ± SD, *p < 0.05, **p < 0.01, ***p < 0.001.

The inflammatory indices (IL-18, IL-1β) in mouse serum were assessed using ELISA. The findings showed notable increases in the inflammatory biomarkers within the CLP mice as opposed to the sham group. However, treatment with XQHF and CA resulted in an improvement in the inflammatory indices in septic mice (**Figures 4A, B**), with more pronounced inflammatory suppression observed in the XQHF group (*p* < 0.001). XQHF demonstrates potential in treating sepsis by reducing pro-inflammatory cytokines and inhibiting inflammatory gene expression and organ damage in CLP mice.

Our analysis of redox enzyme activity in murine lung tissue provided additional evidence that XQHF mitigates inflammation and oxidative stress in CLP-induced mice. The CLP group exhibited significantly higher concentrations of oxidative stress markers—including nitric oxide (NO), myeloperoxidase (MPO), malondialdehyde (MDA), catalase (CAT), and reactive oxygen species (ROS)—along with diminished superoxide dismutase (SOD) activity when compared to the sham group (**Figures 4C–H**). However, XQHF and CA inhibited NO, MPO, MDA, CAT and ROS levels and promoted SOD activity in lung tissues of septic mice. Moreover, XHQF inhibited NO, CAT, and ROS more significantly (*p* < 0.001). This suggests that XQHF has a significant effect on CLP-induced sepsis.

### 3.4 XQHF alleviated NLRP3 inflammasome activation and pyroptosis *in vivo*

**Figure 5:**
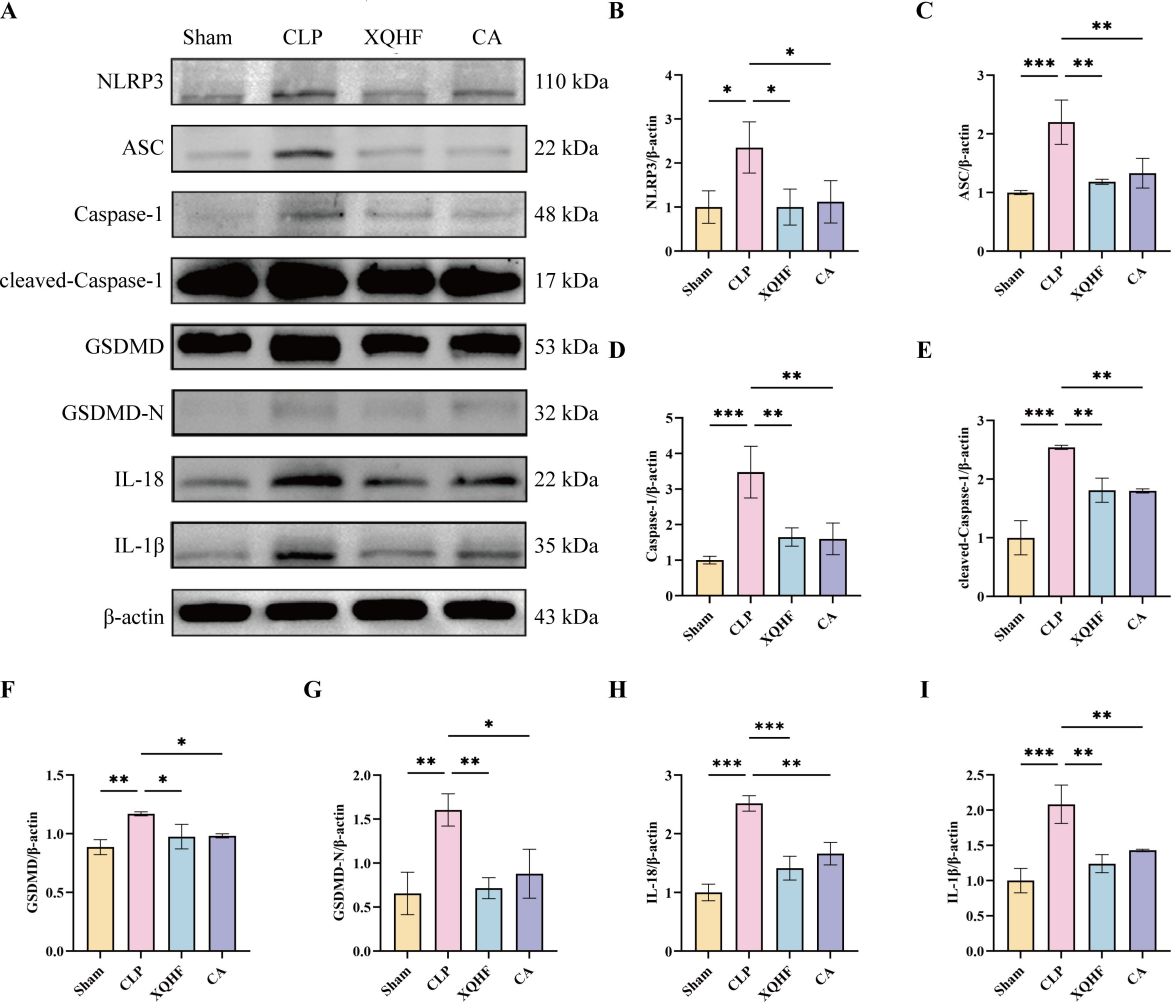
XQHF alleviates NLRP3 inflammasome activation and pyroptosis in CLP-induced sepsis mice. **(A-I)** Protein expressions of NLRP3, ASC, Caspase-1, cleaved-Caspase-1, GSDMD, GSDMD-N, IL-18, and IL-1β in lungs by western blot (n = 3). Values are expressed as mean ± SD, *p < 0.05, **p< 0.01, ***p < 0.001.

Western blot analysis of lung tissue from CLP-induced septic mice unveiled the effect of XQHF on NLRP3 inflammasome triggering. The research found that in the CLP mice, there was a marked increase in the presence of NLRP3, ASC, and downstream signaling molecules like Caspase-1, GSDMD, and its fragment GSDMD-N, as compared to the sham group (**Figure 5A**). Notably, both XQHF and CA significantly reversed these effects (**Figures 5B–I**). It’s noteworthy that XQHF had a more pronounced effect on repressing the expression of GSDMD-N and IL-18 than the CA treatment did. These results suggest that XQHF helps mitigate septic lung damage by downregulating NLRP3 inflammasome-mediated pyroptosis.

### 3.5 XQHF suppressed inflammatory factor release in the LPS + Nig-induced *in vitro*

**Figure 6:**
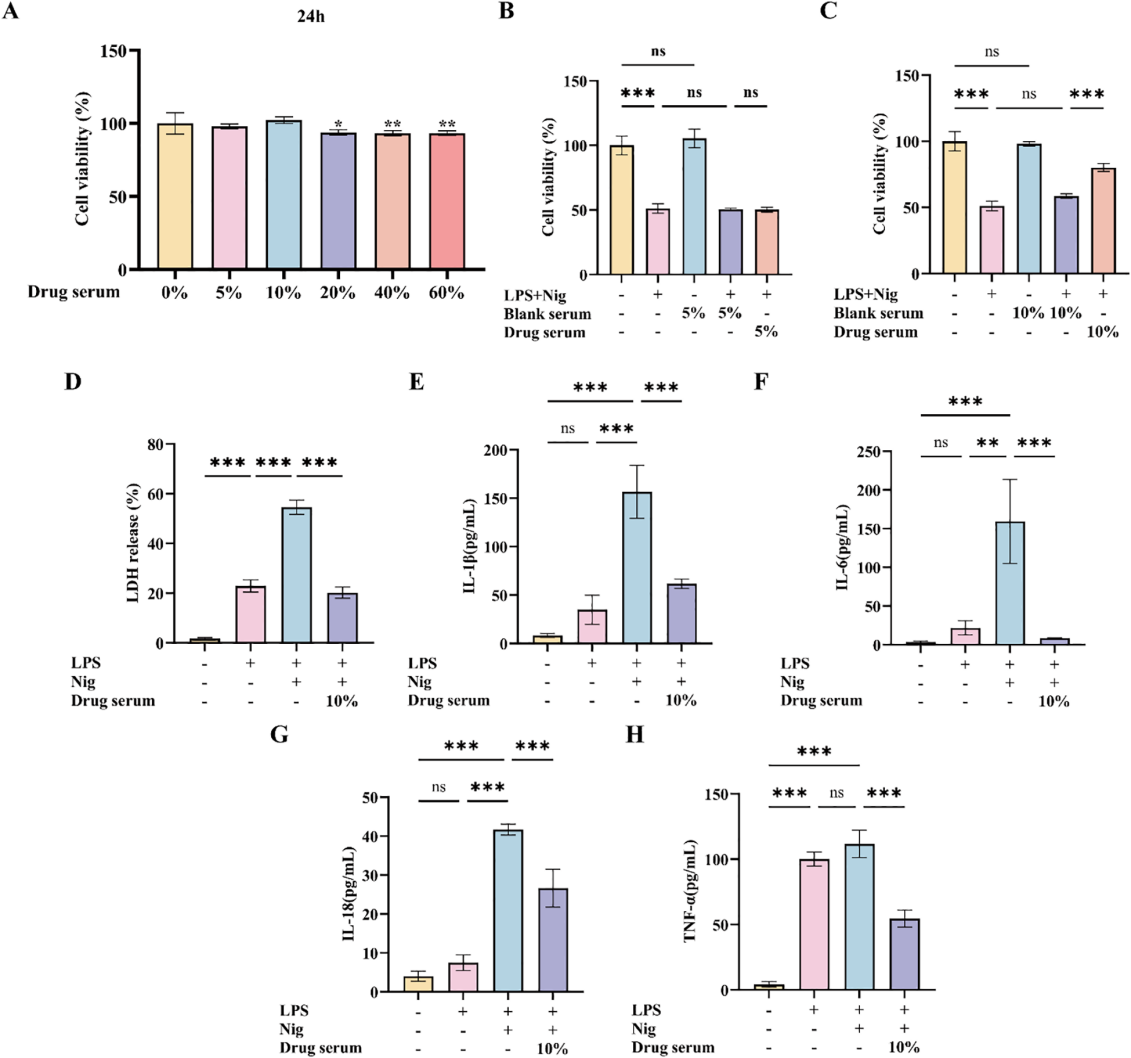
XQHF suppressed inflammatory factor release in classical pyroptosis of LPS + Nig-induced iBMDMs. **(A)** The viability of iBMDMs was determined using the CCK-8 assay after Drug serum challenge for 24 h (n = 5). * *p* < 0.05, ** *p* < 0.01 (vs Blank Control). **(B, C)** CCK-8 assay assessed the cytotoxicity of Blank Control, LPS + Nig, Blank serum, LPS + Nig + Blank serum, and LPS + Nig + Drug serum on iBMDMs (n = 5). **(D)** LDH levels were measured using an LDH assay kit (n = 3). **(E-H)** IL-1β, IL-6, IL-18, and TNF-α levels in culture medium were measured via ELISA (n = 3). Values are expressed as mean ± SD, ns, no significant difference, *p < 0.05, **p < 0.01, ***p < 0.001.

IBMDMs were treated with various concentrations of XQHF-containing serum, and safety was evaluated via CCK-8 assay at 24 h. No significant differences in cell viability were observed between the 5% and 10% XQHF groups and control, while 20%, 40%, and 60% XQHF significantly reduced cell viability (**Figure 6A**). Thus, 5% and 10% XQHF-containing serum were selected for further analyses. Further experiments showed 5%/10% blank serum had no effect on cell viability vs. control, but LPS + Nig treatment significantly reduced viability, confirming successful establishment of the inflammatory pyroptosis model (**Figures 6B, C**, p < 0.001). Additionally, 5%/10% blank serum and 5% XQHF-containing serum did not affect the pyroptosis model (**Figures 6B, C**), whereas 10% XQHF-containing serum significantly reversed LPS + Nig-induced viability reduction (**Figure 6C**, p < 0.001). Therefore, cells in the XQHF group were treated with medium supplemented with 10% XQHF-containing serum, while all other groups received medium containing 10% blank serum.

LDH activity was measured after LPS + Nig stimulation to assess cell death. Both LPS and LPS + Nig increased cell death compared to control and LPS + Nig was more significant (*p* < 0.001). However, iBMDMs treated with 10% drug-containing serum showed a significant reduction in cell death compared to the LPS + Nig group (*p* < 0.001), suggesting that XQHF has a cytoprotective effect on the LPS + Nig-induced classical cellular focal death model (**Figure 6D**). Also, we tested inflammatory factors of the supernatant of treated iBMDMs through ELISA. TNF-α levels rose post-LPS stimulation versus the control group (**Figure 6H**), while IL-1β, IL-6, and IL-18 did not show significant changes. However, compared to LPS stimulation alone, the release of IL-1β, IL-6, and IL-18 significantly increased after LPS + Nig stimulation (**Figures 6E-G**, p < 0.01, *p* < 0.001), whereas in cells treated with 10% drug-containing serum, the levels of these cytokines in the supernatant were severely suppressed compared to the LPS + Nig group (*p* < 0.001). The results indicate that XQHF suppresses the expression of inflammatory factors in the classical focused killing of macrophages triggered by LPS + Nig.

### 3.6 XQHF ameliorated pyroptosis of LPS + Nig-induced *in vitro*

**Figure 7:**
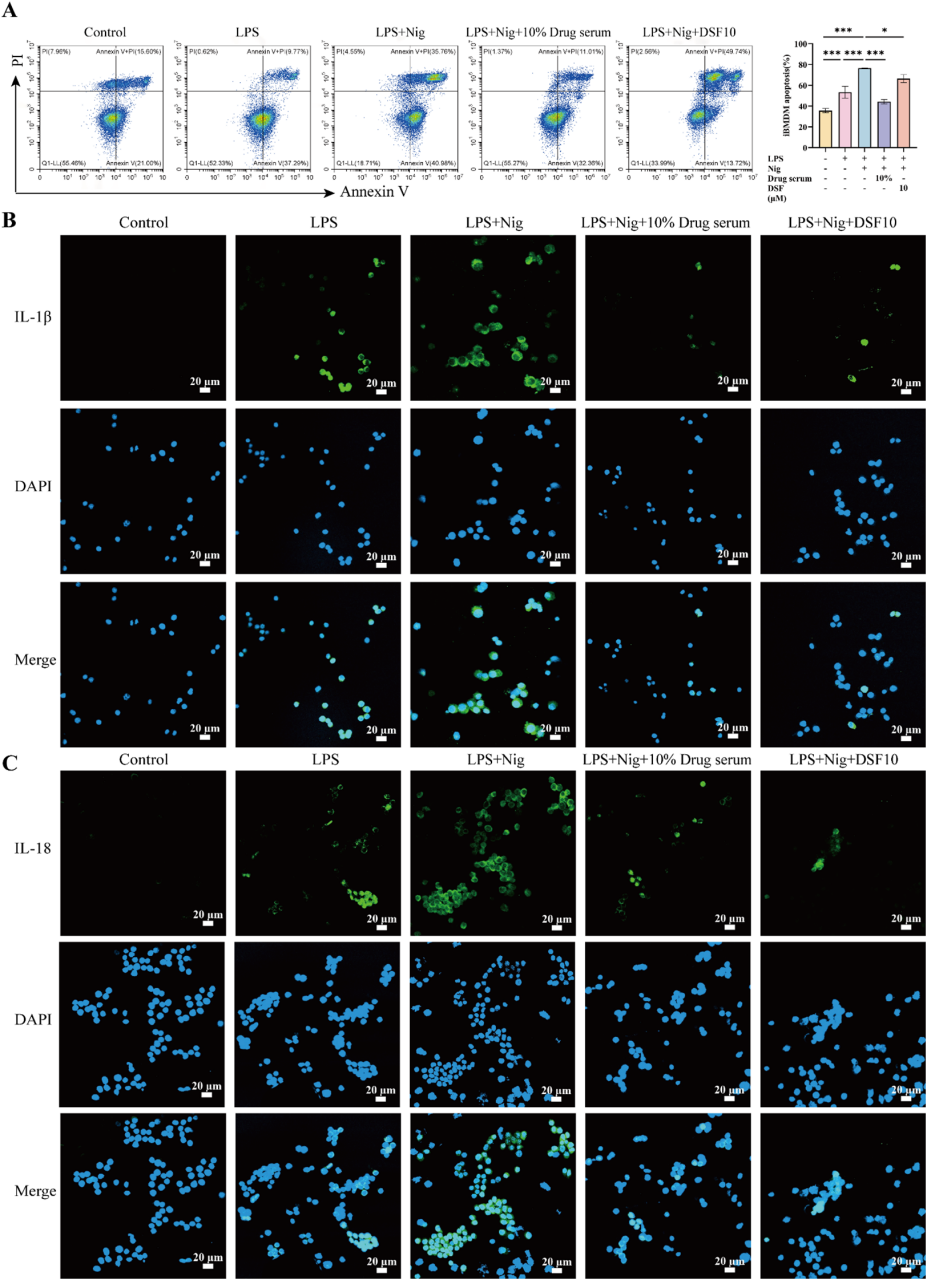
XQHF ameliorated classical pyroptosis of LPS + Nig-induced iBMDMs. **(A)** Flow cytometry was conducted to detect apoptosis (n = 3). **(B, C)** IL-1β and IL-18 in iBMDMs measured by immunofluorescence staining showed green fluorescence (n = 3). Scale bars, 20 μm. Values are expressed as mean ± SD, *p < 0.05, **p < 0.01, ***p < 0.001.

In flow cytometry, by comparing the overall early and late apoptosis rates among different groups of cells, it was showed that both LPS stimulation and LPS + Nig stimulation enhanced apoptosis compared to controls, and apoptosis induced by LPS + Nig stimulation was more pronounced. In contrast, XQHF and DSF reversed this outcome, and surprisingly, XQHF treatment attenuated apoptosis more significantly (**Figure 7A**, p < 0.001).

The immunofluorescence analysis revealed a marked rise in iBMDMs recruitment following both LPS stimulation alone and LPS combined with Nig treatment compared to the control. Additionally, the classical pyroptosis model of LPS + Nig-induced iBMDMs exhibited substantially elevated levels of IL-1β and IL-18 expression under these conditions (**Figures 7B, C**). But XQHF significantly reduced their expression, aligning with the *in vitro* findings, while DSF exhibited a similar effect. These results support the conclusion that XQHF ameliorates cellular pyroptosis and thus reduces inflammation.

### 3.7 XQHF inhibited NLRP3/Caspase-1 signaling pathway *in vitro*

**Figure 8:**
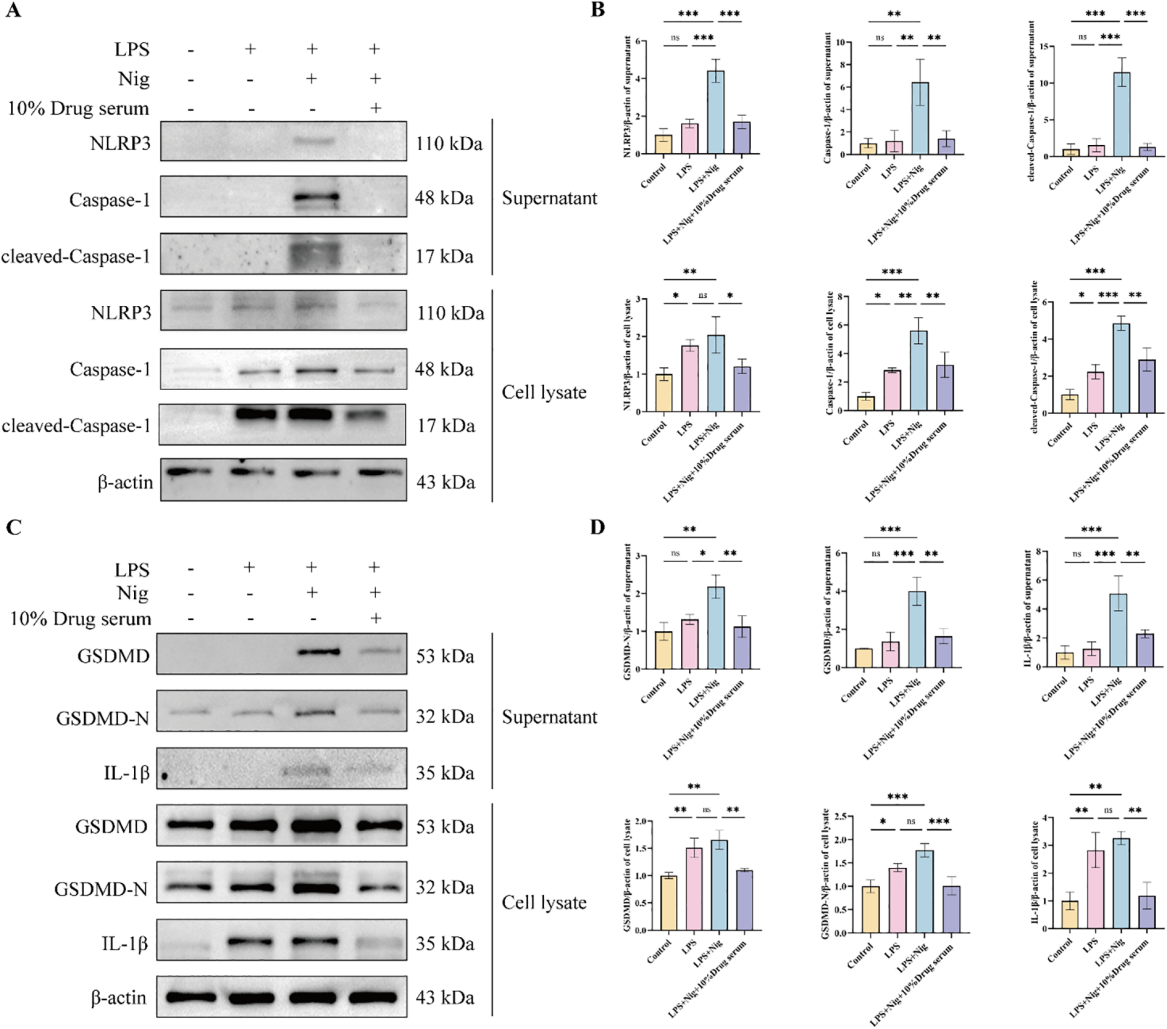
XQHF inhibited NLRP3/Caspase-1 signaling pathway and NLRP3 inflammasome of classical pyroptosis of LPS + Nig-induced iBMDMs. **(A)** NLRP3, Caspase-1, and cleaved-Caspase-1 were identified through western blot (n = 3). **(B)** NLRP3, Caspase-1 and cleaved-Caspase-1 protein expression (n = 3). **(C)** The protein expression of GSDMD, GSDMD-N and IL-1β were detected by western blotting (n = 3). **(D)** GSDMD, GSDMD-N and IL-1β protein expression (n = 3). β-actin was used as an internal control. Values are expressed as mean ± SD, ns, no significance, *p < 0.05, **p < 0.01, ***p < 0.001.

By modeling LPS + Nig-induced classical pyroptosis in macrophages, we observed whether the results in cellular proteins and cell supernatants were consistent. The data showed that, compared with the LPS+Nig group, XQHF induced the downregulation of NLRP3, Caspase-1, and cleaved-Caspase-1 expression (**Figures 8A, B**). Furthermore, XQHF significantly inhibited GSDMD protein cleavage and reduced IL-1β release and expression. (**Figures 8C, D**). These findings suggest that XQHF inhibits the expression of factors associated with the NLRP3/Caspase-1 pathway and suppresses NLRP3 inflammatory activation.

## 4 Discussions

ALI is not only a public health problem of global concern, but also a considerable economic burden for countries worldwide. Due to the lack of specific drugs to treat sepsis, sepsis-related morbidity and mortality continue to increase during clinical interventions (20). Herbal medicines derived from natural sources offer the benefit of fewer side effects and the potential for multi-targeted therapy in the treatment of sepsis. Recent research indicates that herbal decoctions demonstrate superior efficacy in treating ALI in sepsis (12, 21, 22).

XQHF is a representative formula of Xuan-transparency and heat-clearing, conciliation, and spleen-strengthening, which has achieved good results in treating of ALI in clinical practice. We identified various active ingredients of XQHF by UPLC-MS/MS, including Wogonoside, Baicalin, Apigenin 7-O-beta-D-glucuronide, Forsythoside, and CA. It was found that Forsythoside A (FA), an extract of *Forsythia suspensa*, ameliorated LPS-induced ALI pathology, an increase in lung water content and inflammatory cytokines, cellular infiltration, and activation of the STAT3 signaling pathway in BALB/c mice, thus alleviating septic lung injury (23). Recent investigations indicate that substances such as Wogonoside and CA, derived from herbal decoctions, safeguard macrophages from normal Nigerian bacteriocin-induced and cytoplasmic LPS-induced atypical cellular charring, while also mitigating LPS-induced septic mice (24, 25). We hypothesized that XQHF may ameliorate septic lung injury by improving cellular focal death through multiple components and targets. The current investigation demonstrated that treatment with XQHF-H significantly enhanced the 7-day survival rate of mice with CLP-induced ALI. Additionally, alterations in H&E staining, decreased lung W/D ratios, and lower BALF protein levels, demonstrated that XQHF therapy effectively alleviated pulmonary edema, hemorrhage, and inflammatory cell accumulation in CLP-induced septic mice. The results emphatically indicate that XQHF exerts protective effects on lung tissue during sepsis.

Multiple studies pinpoint NLRP3 as a crucial therapeutic target to mitigate sepsis-related inflammation and organ dysfunction (3, 26, 27). The NLRP3 inflammasome activates when NLRP3, ASC, and pro-caspase-1 form a complex, triggering IL-1β release. This process promotes inflammation and pyroptosis in ALI and ARDS (28). Macrophages dominate the immune cell population within lung tissue, serving as critical guardians of tissue balance by triggering defensive mechanisms when pathogens are detected. That said, an overactive inflammatory reaction can spiral out of control, ultimately contributing to acute lung injury and acute respiratory distress syndrome. *Wang* et al. found that blocking lung macrophage accumulation reduces IL-27 production, lowering TNF-α and IL-1β levels, which can alleviate sepsis-related lung damage (29). NLRP3 inflammatory-mediated IL-1β cleavage and cellular pyroptosis are pivotal in the progression of ALI/ARDS. NLRP3 cleaves Caspase-1, which cleaves GSDMD and releases IL-1β and IL-18 from inflammatory factors. Inflammatory response also damages pulmonary vascular endothelial cells and attracts many immune cells into the lung tissues, aggravating the lung injury. Oxidative stress also exacerbates the inflammatory response. Antioxidant defenses safeguard biological systems from free radical toxicity. TCM is effective in anti-inflammatory treatment. It was shown that Shenfu Decoction enhanced neutrophil chemotaxis and regulated anti-inflammatory activity in CLP-induced sepsis mice (30). Wei et al. showed that Gegen Qinlian pills significantly reduced TNF-α and IL-1β expression in an *in vitro* LPS-induced cell model (31). Our *in vivo* results confirmed that XQHF, as an innovative intervention for the treatment of sepsis, has shown a pronounced effect in reducing the release of pro-inflammatory cytokines in blood serum while simultaneously lowering oxidative stress markers in lung tissue from CLP mice, a finding of significant academic interest and clinical relevance. The *in vitro* studies further demonstrated XQHF’s dual action—it suppressed both inflammatory mediators (IL-18, IL-1β, IL-6, and TNF-α) and prevented programmed cell death in LPS + Nig-induced iBMDM cells. These findings collectively highlight XQHF’s therapeutic potential in modulating inflammatory responses. Pyroptosis shares a close connection with apoptosis, although these cellular processes are not entirely distinct from one another. Research has identified GSDME and GSDMD as crucial regulators shifting apoptosis to pyroptosis. Additionally, studies reveal a functional interplay between Caspase-3 and Caspase-1—the primary effector enzymes responsible for executing apoptosis and pyroptosis, respectively. This molecular crosstalk highlights the interconnected nature of these programmed cell death pathways (32). The NLRP3 inflammasome facilitates apoptosis through its enhancement of IL-1β and IL-18 secretion. On the contrary, the prevention of NLRP3 inflammasome activation inhibits apoptosis (33). Chen et al. discovered that calycosin, a compound derived from the roots of *Astralagus membranaceus*, was effective in lowering apoptosis rates. It also demonstrated anti-inflammatory properties, successfully inhibited the NLRP3 inflammasome from activating, bolstered lung function in young septic rats, and ultimately mitigated the severity of ALI caused by sepsis (34). Simultaneously, Zhang et al. found that CB2 receptor activation mitigated apoptosis, enhanced cell survival, and decreased NLRP3 inflammasome activity, thereby lessening sepsis-related lung injury (35). Therefore, combining our results, XQHF can inhibit pyroptosis of the NLRP3/Caspase-1 pathway, helping to mitigate lung injury.

In summary, our investigation of XQHF exhibits certain shortcomings, including a lack of additional testing on the active ingredients, a focus on short-term efficacy, and an absence of assessment regarding the sustained effectiveness and safety of the Chinese herbal remedy. In the subsequent phase, we will persist in integrating multi-omics technologies to investigate the mechanistic implications of XQHF further.

## 5 Conclusion

This study investigates the potential mechanisms of XQHF in the treatment of sepsis-related ALI. *In vitro* and *in vivo* results indicate that XQHF inhibits the NLRP3/Caspase-1-mediated pyroptosis pathway, reducing macrophage pyroptosis and decreasing the release of pro-inflammatory factors IL-1β and IL-18, thereby alleviating lung tissue damage and improving survival rates in septic mice. In summary, XQHF may exert protective effects on the lungs by inhibiting NLRP3 inflammasome activation, lowering the expression of Caspase-1 and GSDMD, and blocking the pyroptosis cascade, leading to reduced inflammation and cytokine storms. Additionally, Wogonoside, Baicalin, and CA are likely the major active components. This study provides theoretical and experimental evidence for the application of XQHF as an NLRP3 inflammasome inhibitor in the treatment of ALI.

## Data Availability

The raw data supporting the conclusions of this article will be made available by the authors, without undue reservation.
