# Supplementary material for: Xuanqing Hefa formula relieves sepsis-triggered acute lung injury by targeting the NLRP3/Caspase-1 pyroptosis mechanism
Source: Front Immunol. 2026 Feb 3;16:1709586. doi: 10.3389/fimmu.2025.1709586 (PMC12910159; doi:10.3389/fimmu.2025.1709586)

Supplementary files

Fig.3C

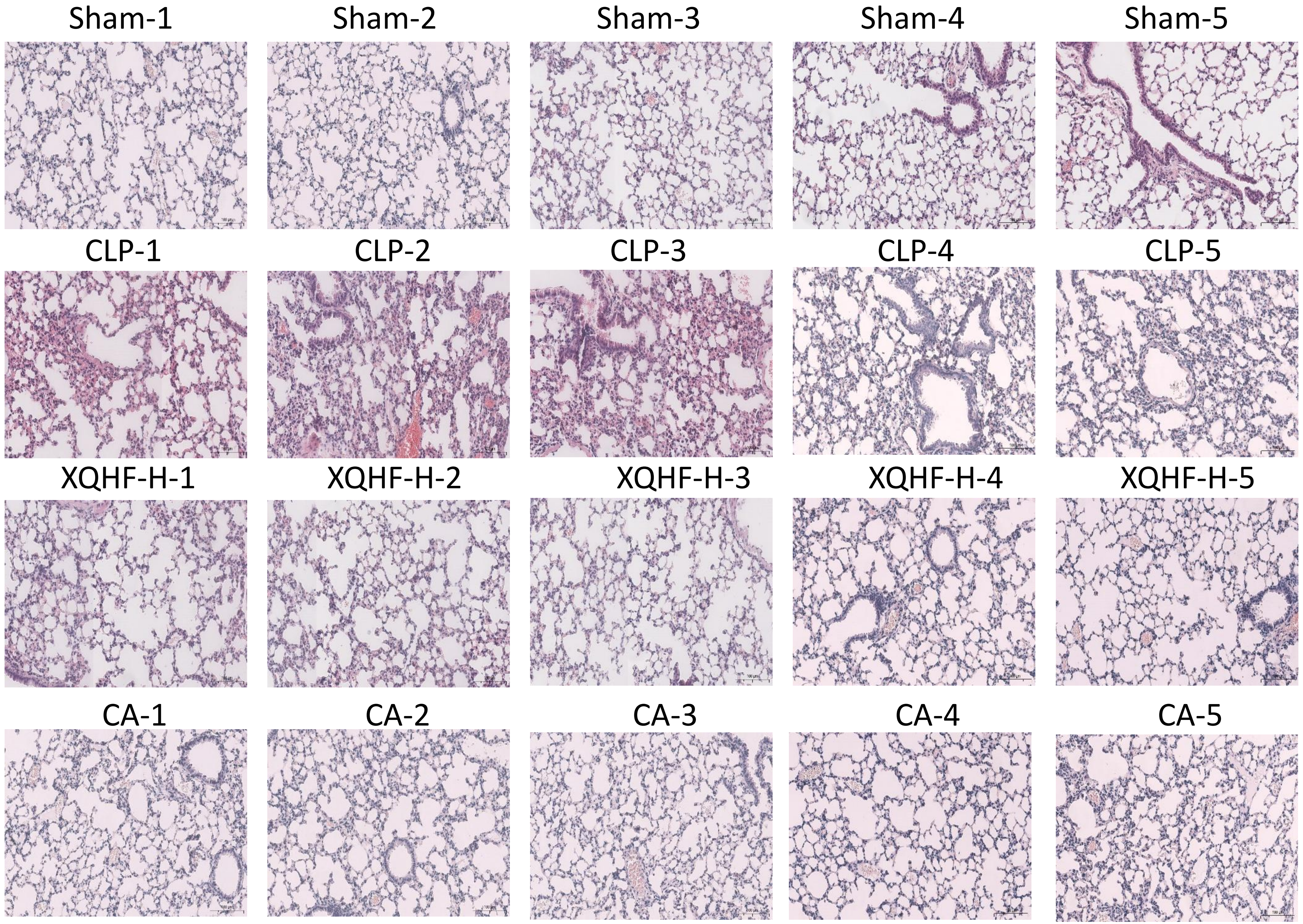
Sham-1
Sham-2
Sham-3
Sham-4
Sham-5
CLP-1
CLP-2
CLP-3
CLP-4
CLP-5
XQHF-H-1
XQHF-H-2
XQHF-H-3
XQHF-H-4
XQHF-H-5
CA-1
CA-2
CA-3
CA-4
CA-5

Fig.3D-F

Lung injury score

Sham	CLP	XQHF-H	CA
0	4	1	1
0	4	1	1
0	3	2	2
0	4	2	3
0	3	2	2

W/D ratio of lung

Sham	CLP	XQHF	CA
3.405345	5.349693	3.855263	4.721116
3.508658	5.519774	3.914005	4.728358
3.705314	7.613139	3.915212	4.762774
3.73399	8.151163	3.980435	4.882096
3.737452	8.317568	4.05641	4.890196

Protein in BALF

Sham	CLP	XQHF-H	CA
70.41946	230.8161	141.3996	193.9433
73.18492	235.4252	146.0087	198.5524
103.605	236.3471	151.5396	200.3961
110.9795	237.2689	156.1487	212.3797
125.7287	241.878	198.5524	176.4288

Fig.4A-D

IL-18

Sham	CLP	XQHF	CA
4.115921	29.5294	15.80457	27.77095
6.376738	29.96845	16.25084	29.09013
7.730223	33.90978	16.69687	22.91632
8.180887	34.34659	17.14266	25.56775
9.981065	36.09164	17.5882	27.33077

IL-1β

Sham	CLP	XQHF	CA
6.86797	49.2419	11.7478	25.79058
8.561211	51.50184	14.16106	28.75821
9.689148	61.84116	15.31828	30.51998
10.64614	105.4346	15.88023	33.7735
10.81638	109.7398	16.44202	34.32994

NO

Sham	CLP	XQHF	CA
3.200755	6.514129	4.335671	5.524412
3.334315	6.542758	4.525191	5.536819
3.542299	7.558153	4.690084	5.714981
3.639028	9.185845	4.763271	5.718605
3.648215	10.86862	4.780835	5.816833

SOD

Sham	CLP	XQHF	CA
1.88591	0.699029	1.3857955	1.014899
1.937844	0.743028	1.4821381	1.184065
1.962134	0.842105	1.4849915	1.229557
1.976383	0.917025	1.6858965	1.32776
1.872189	0.969179	0.9751693	1.697671

Fig.4E-H

MPO

Sham	CLP	XQHF	CA
0.131189	0.267835	0.203081	0.21284
0.126399	0.297286	0.230978	0.193614
0.121406	0.269645	0.222937	0.235028
0.140576	0.230978	0.2017	0.250138
0.141661	0.231067	0.202975	0.199497

MDA

Sham	CLP	XQHF	CA
3.007375	8.704283	4.366431	4.822903
1.79629	7.961722	4.513532	5.005669
2.472302	5.615013	4.631784	5.45351
3.082486	7.866091	3.391051	4.021016
3.262629	11.61039	7.049548	3.375679

CAT

Sham	CLP	XQHF	CA
0.208459	0.95596	0.479553	0.735611
0.281097	0.965872	0.537781	0.773444
0.302632	1.032352	0.582869	0.845308
0.313586	1.062449	0.63693	0.872571
0.326026	1.080802	0.72837	0.941797

ROS

Sham	CLP	XQHF	CA
15255.8	37172.58	22463.62	26713.76
15436.04	46312.79	22491.21	30278.51
16159.89	47085.45	22674.52	31535.38
17053.55	52182.52	24038.94	37916.01
17746.99	31703.44	25252.54	38269.4

Fig.5 Original blots

NLRP3

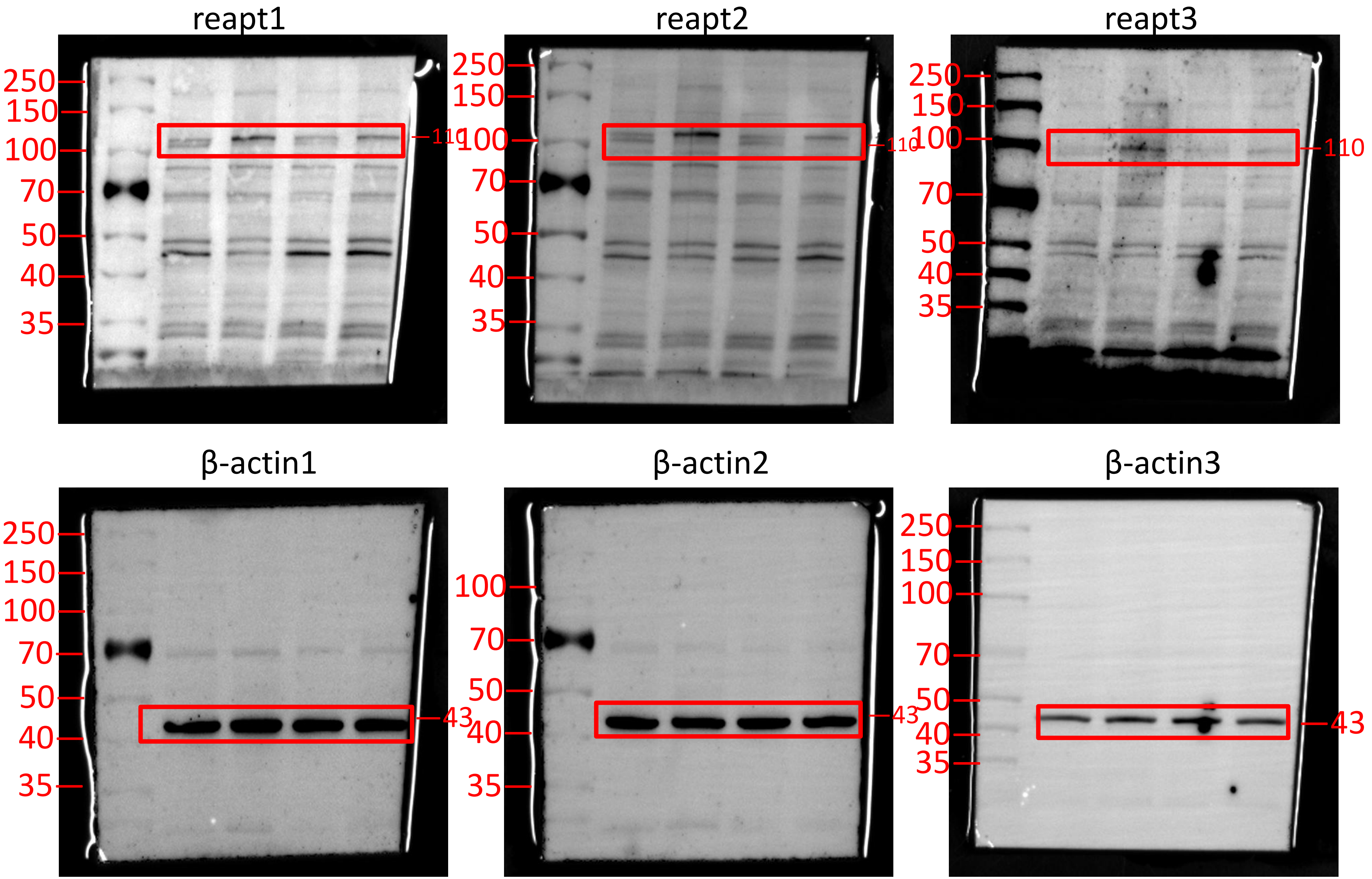

Fig.5 Original blots

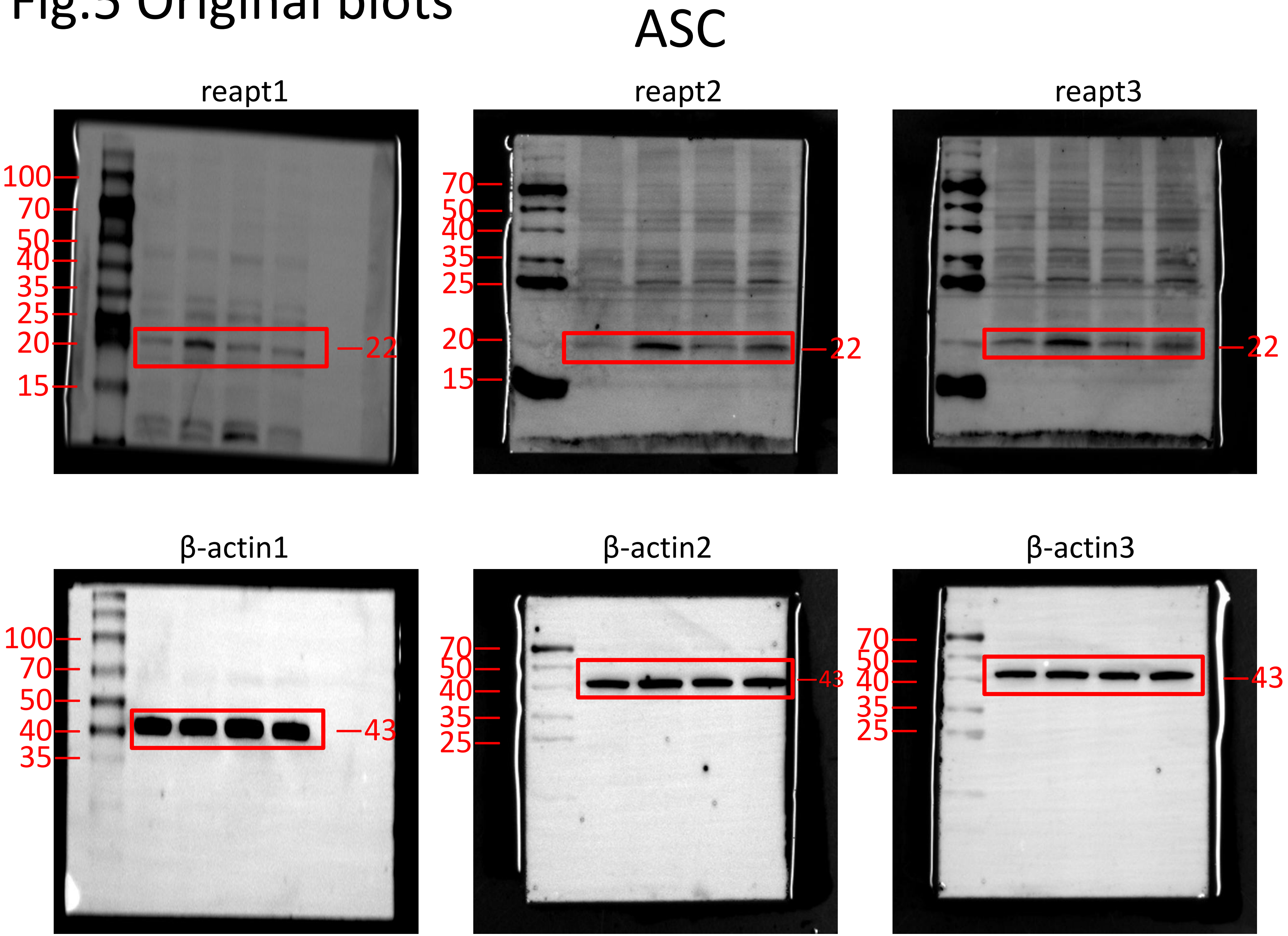

Fig.5 Original blots

Caspase-1 and cleaved-Caspase-1

reapt1

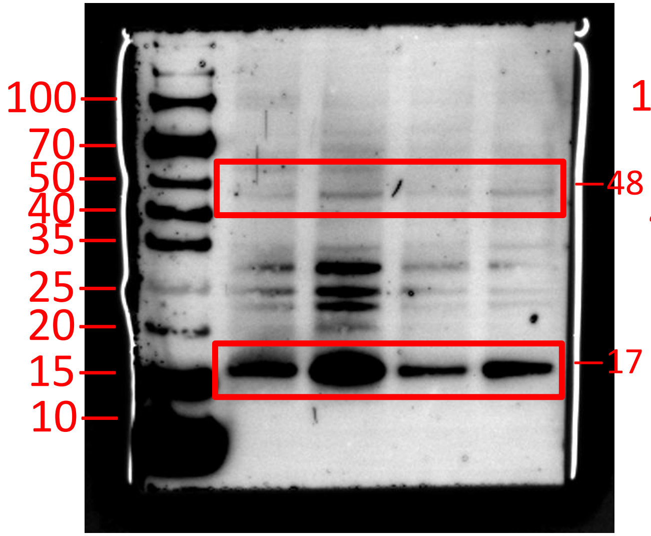

reapt2

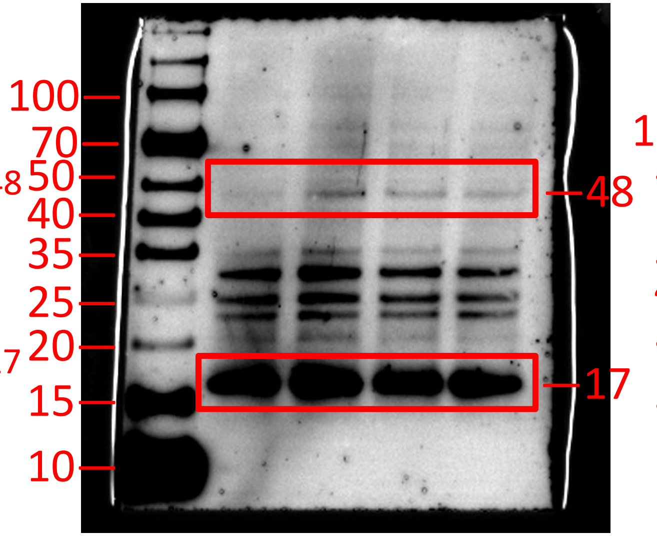

reapt3

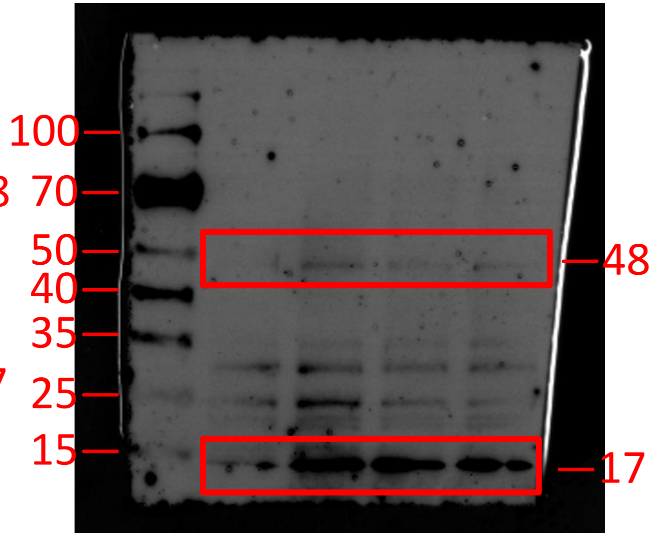

β-actin1

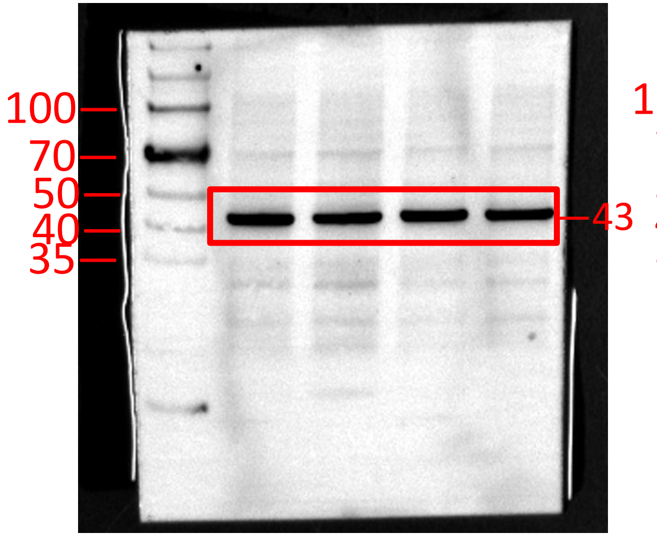

β-actin2

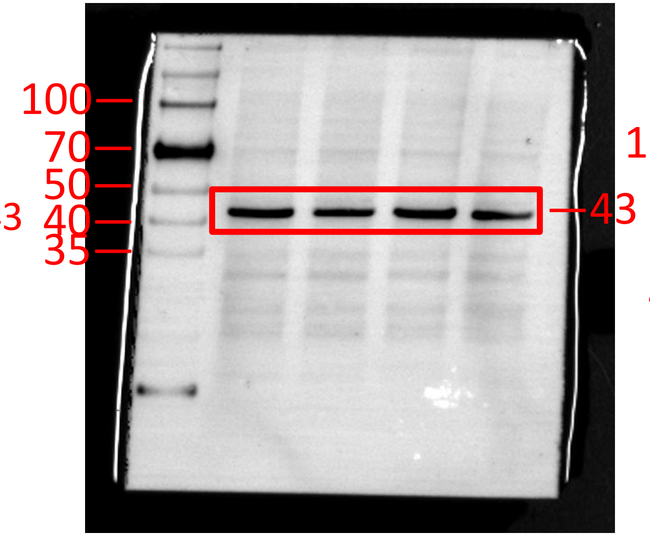

β-actin3

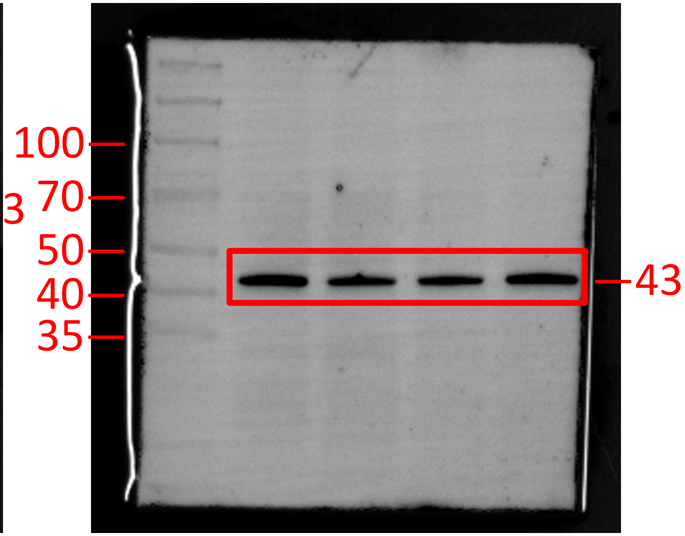

Fig.5 Original blots

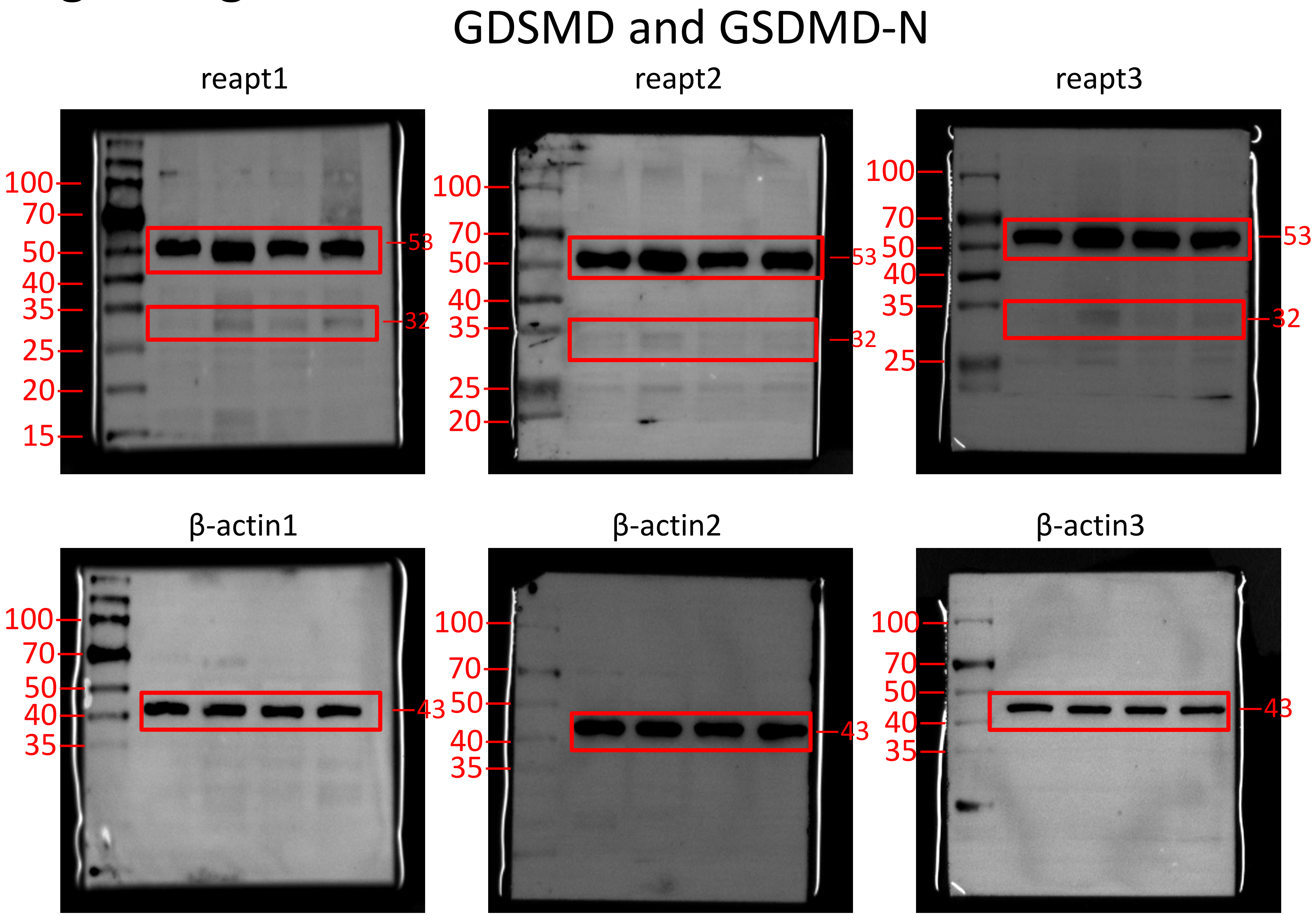

Fig.5 Original blots

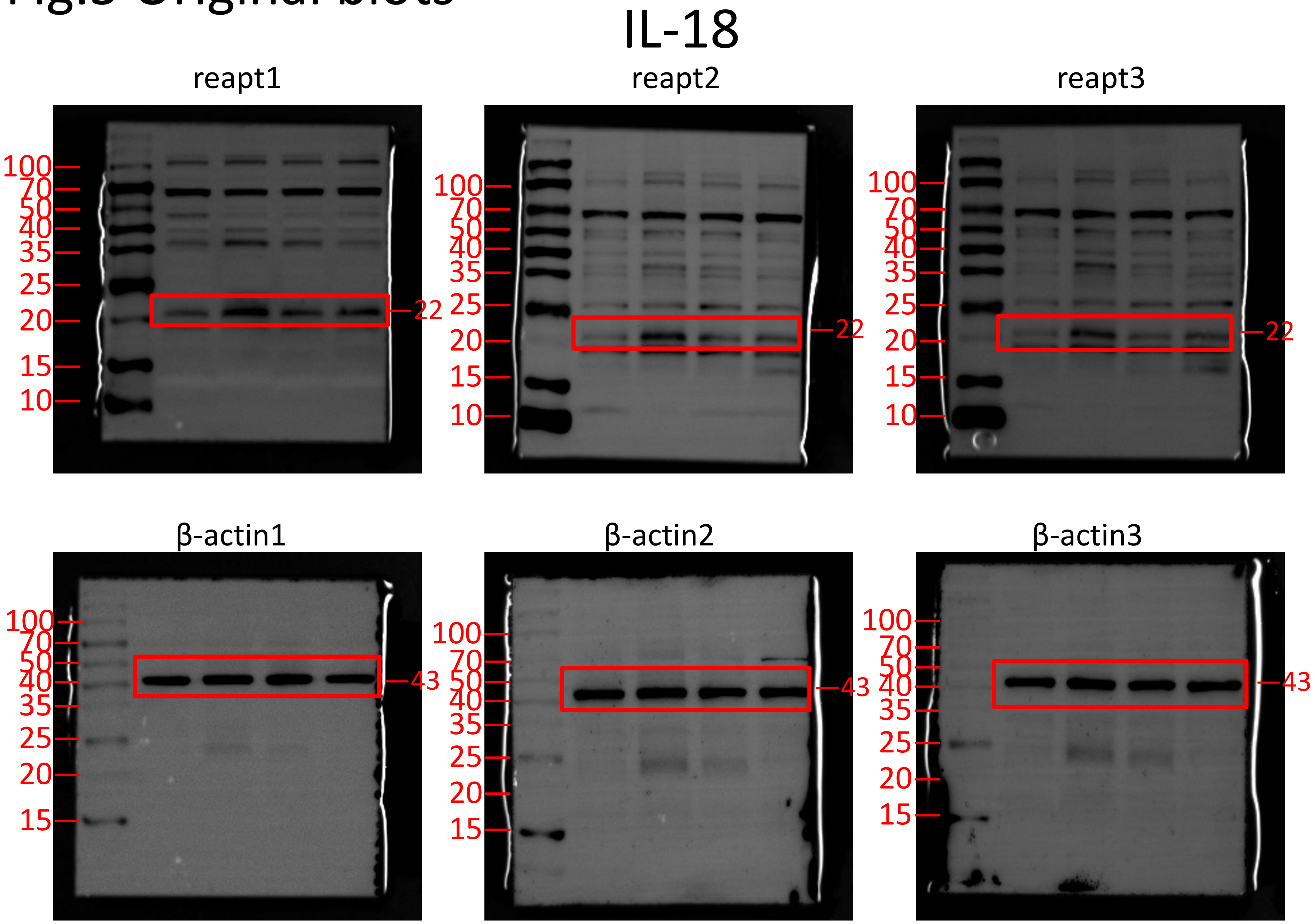

Fig.5 Original blots

IL-1β

reapt1

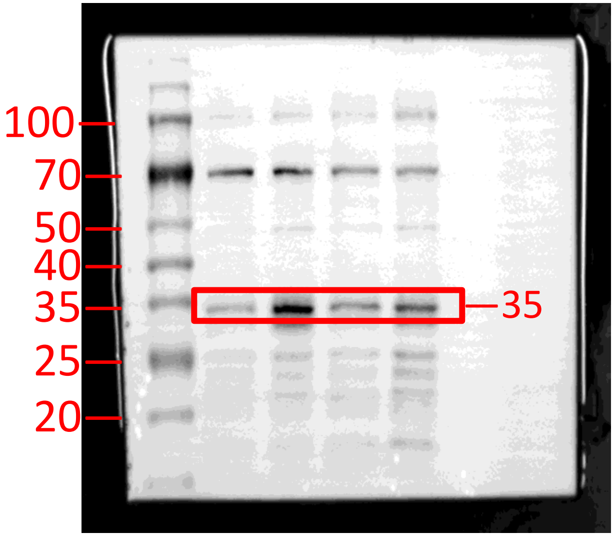

reapt2

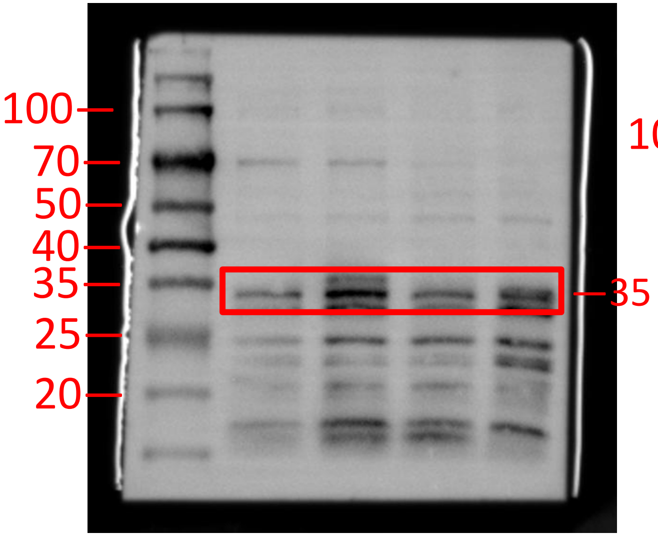

reapt3

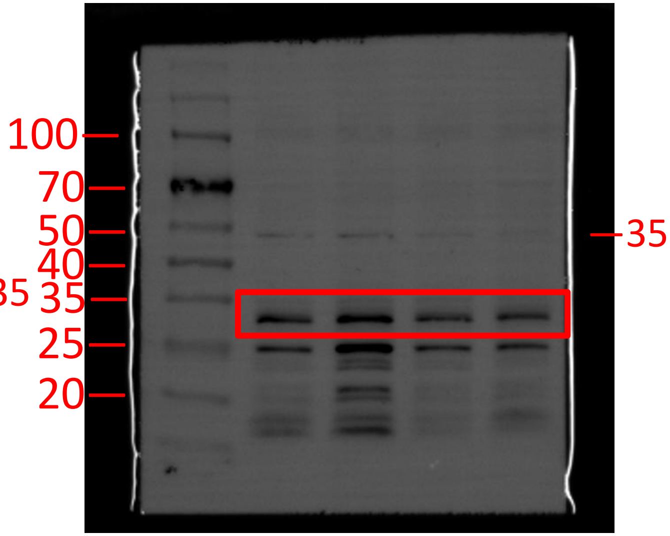

β-actin1

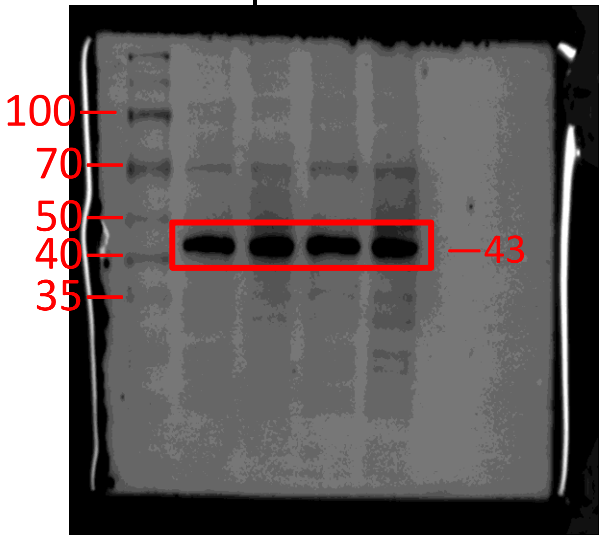

β-actin2

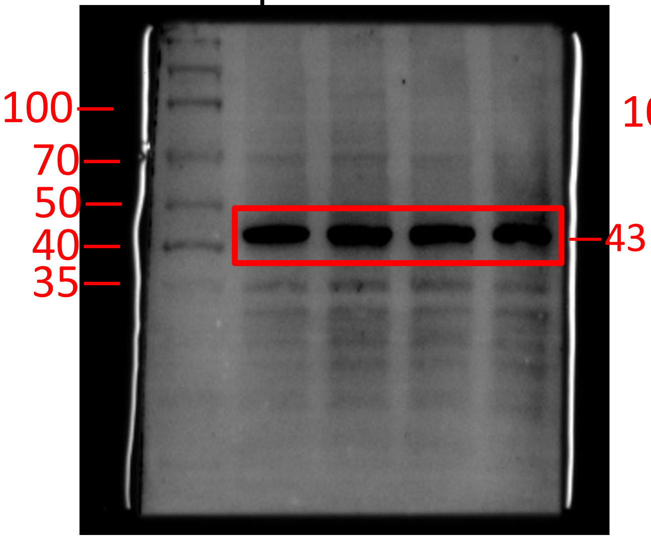

β-actin3

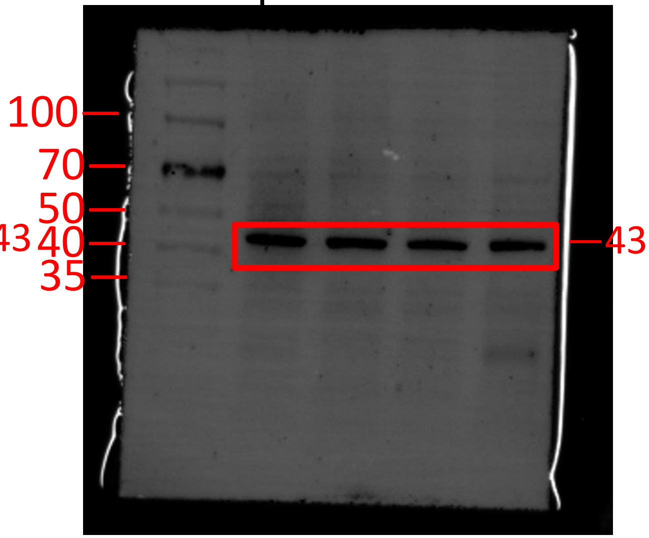

Fig.7A

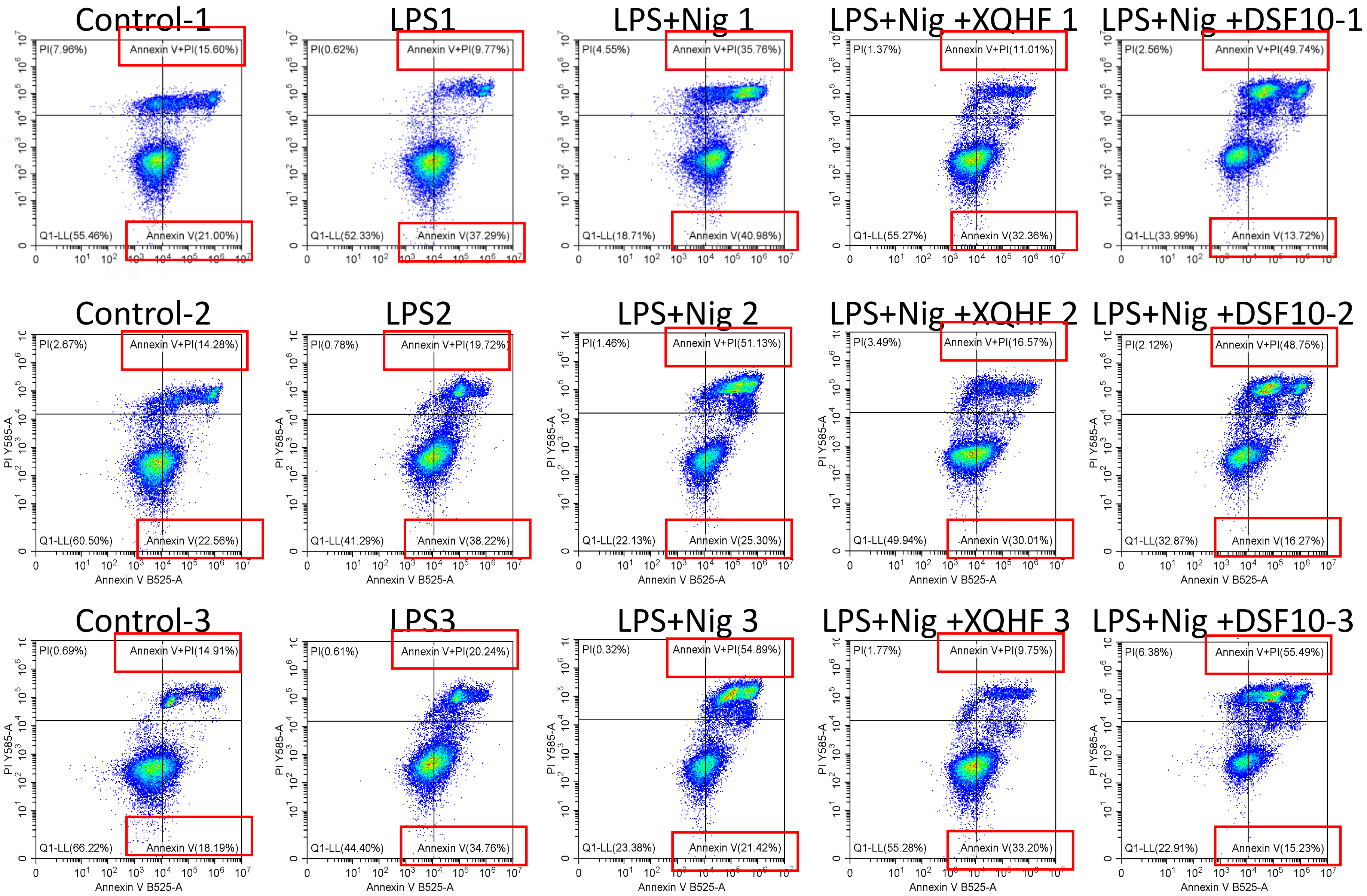

Control-1
PI(7.96%)
Annexin V+PI(15.60%)
Q1-LL(55.46%)
Annexin V(21.00%)
LPS1
PI(0.62%)
Annexin V+PI(9.77%)
Q1-LL(52.33%)
Annexin V(37.29%)
LPS+Nig 1
PI(4.55%)
Annexin V+PI(35.76%)
Q1-LL(18.71%)
Annexin V(40.98%)
LPS+Nig +XQHF 1
PI(1.37%)
Annexin V+PI(11.01%)
Q1-LL(55.27%)
Annexin V(32.36%)
LPS+Nig +DSF10-1
PI(2.56%)
Annexin V+PI(49.74%)
Q1-LL(33.99%)
Annexin V(13.72%)
Control-2
PI(2.67%)
Annexin V+PI(14.28%)
Q1-LL(60.50%)
Annexin V(22.56%)
LPS2
PI(0.78%)
Annexin V+PI(19.72%)
Q1-LL(41.29%)
Annexin V(38.22%)
LPS+Nig 2
PI(1.46%)
Annexin V+PI(51.13%)
Q1-LL(22.13%)
Annexin V(25.30%)
LPS+Nig +XQHF 2
PI(3.49%)
Annexin V+PI(16.57%)
Q1-LL(49.94%)
Annexin V(30.01%)
LPS+Nig +DSF10-2
PI(2.12%)
Annexin V+PI(48.75%)
Q1-LL(32.87%)
Annexin V(16.27%)
Control-3
PI(0.69%)
Annexin V+PI(14.91%)
Q1-LL(66.22%)
Annexin V(18.19%)
LPS3
PI(0.61%)
Annexin V+PI(20.24%)
Q1-LL(44.40%)
Annexin V(34.76%)
LPS+Nig 3
PI(0.32%)
Annexin V+PI(54.89%)
Q1-LL(23.38%)
Annexin V(21.42%)
LPS+Nig +XQHF 3
PI(1.77%)
Annexin V+PI(9.75%)
Q1-LL(55.28%)
Annexin V(33.20%)
LPS+Nig +DSF10-3
PI(6.38%)
Annexin V+PI(55.49%)
Q1-LL(22.91%)
Annexin V(15.23%)
PI Y585-A
Annexin V B525-A

Fig.7A

iBMDM apoptosis(%) （Early Apoptosis + Late Apoptosis）

Control	LPS	LPS+Nig	LPS+Nig+10% Drug serum	LPS+Nig +DSF 10
36.6	47.06	76.31	43.37	63.46
36.84	55	76.43	42.95	65.02
33.1	57.94	76.74	46.58	70.72

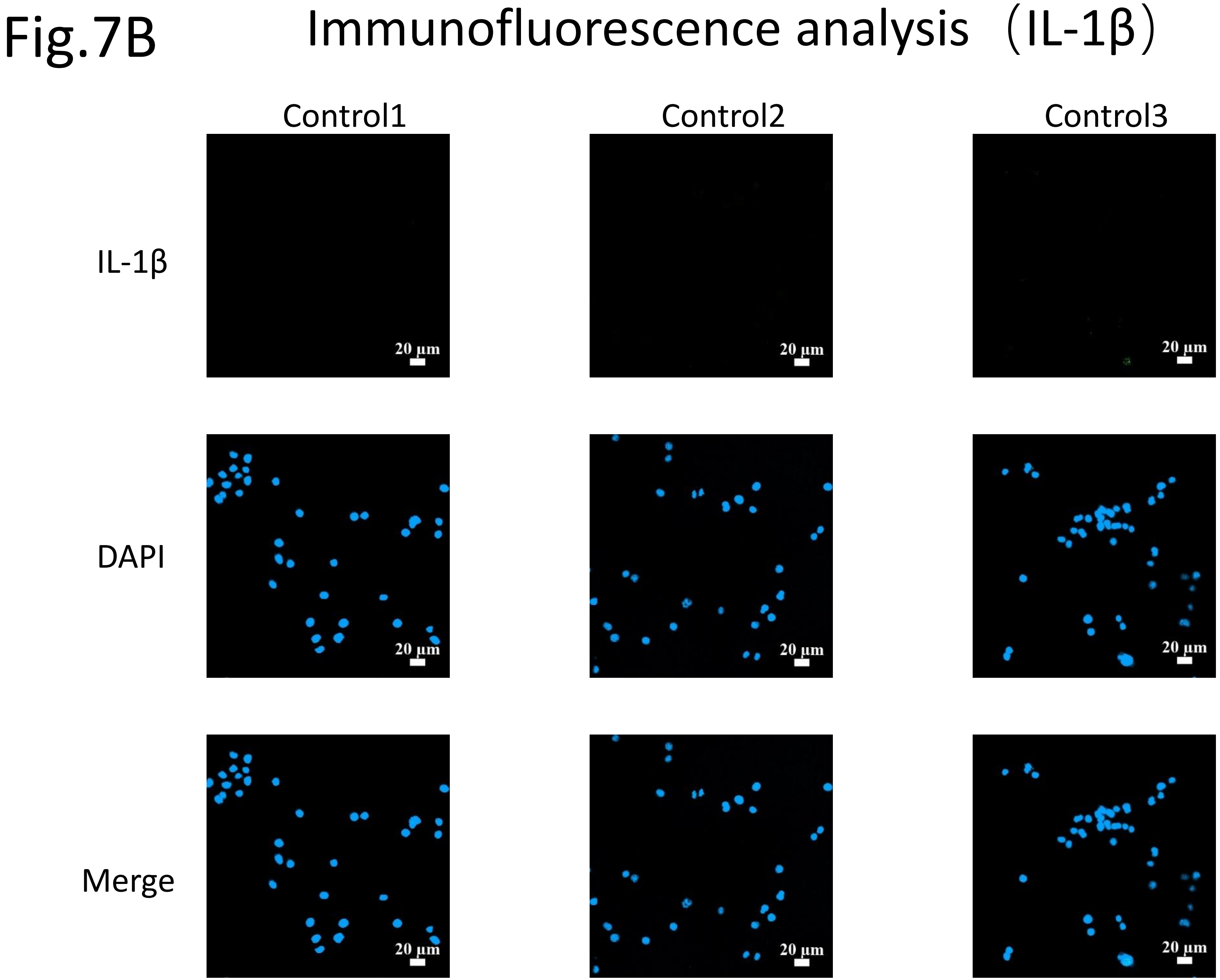
Fig.7B
Immunofluorescence analysis (IL-1β)
Control1
Control2
Control3
IL-1β
DAPI
Merge
20 μm

Fig.7B

Immunofluorescence analysis (IL-1β)

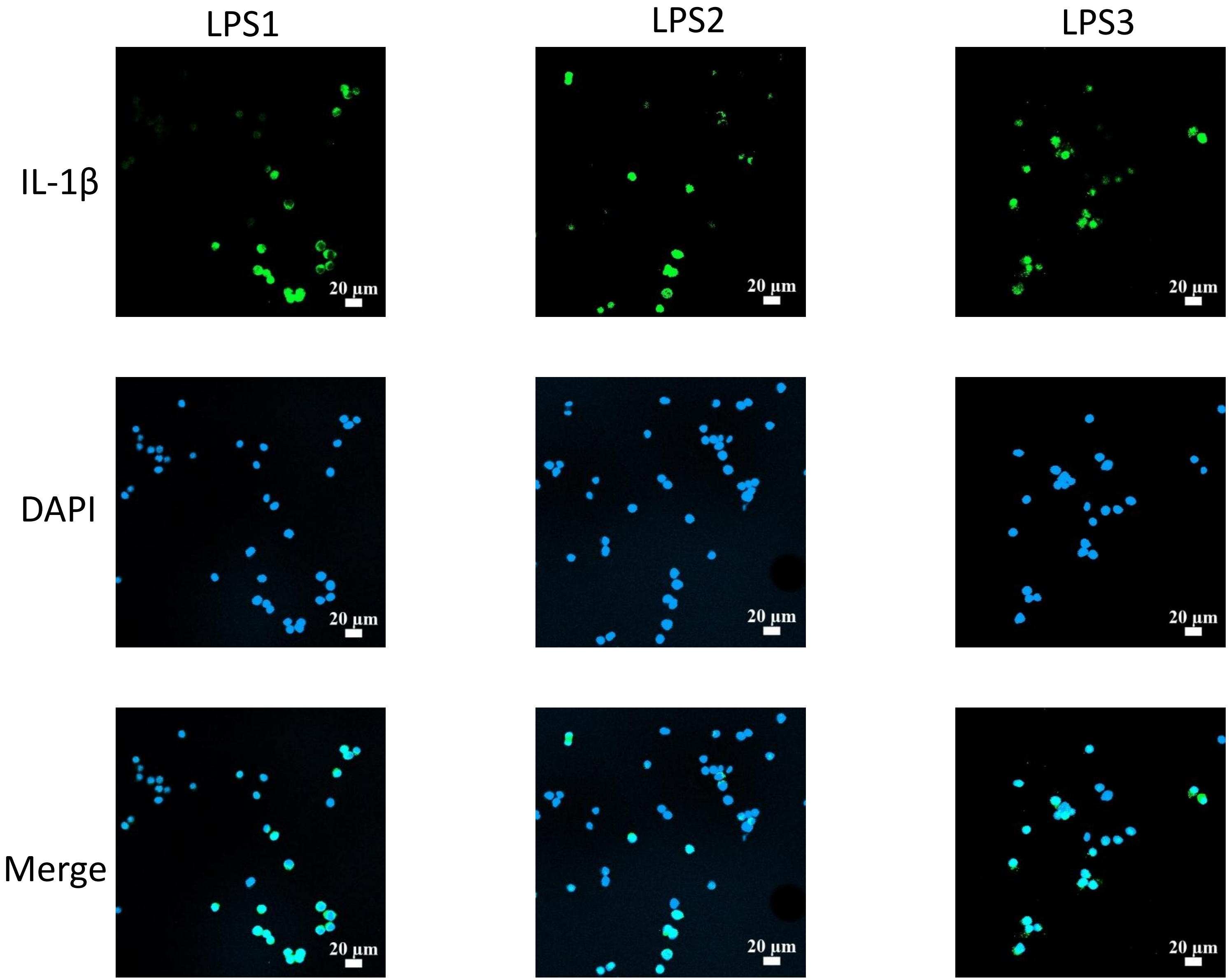

Fig.7B

Immunofluorescence analysis（IL-1β）

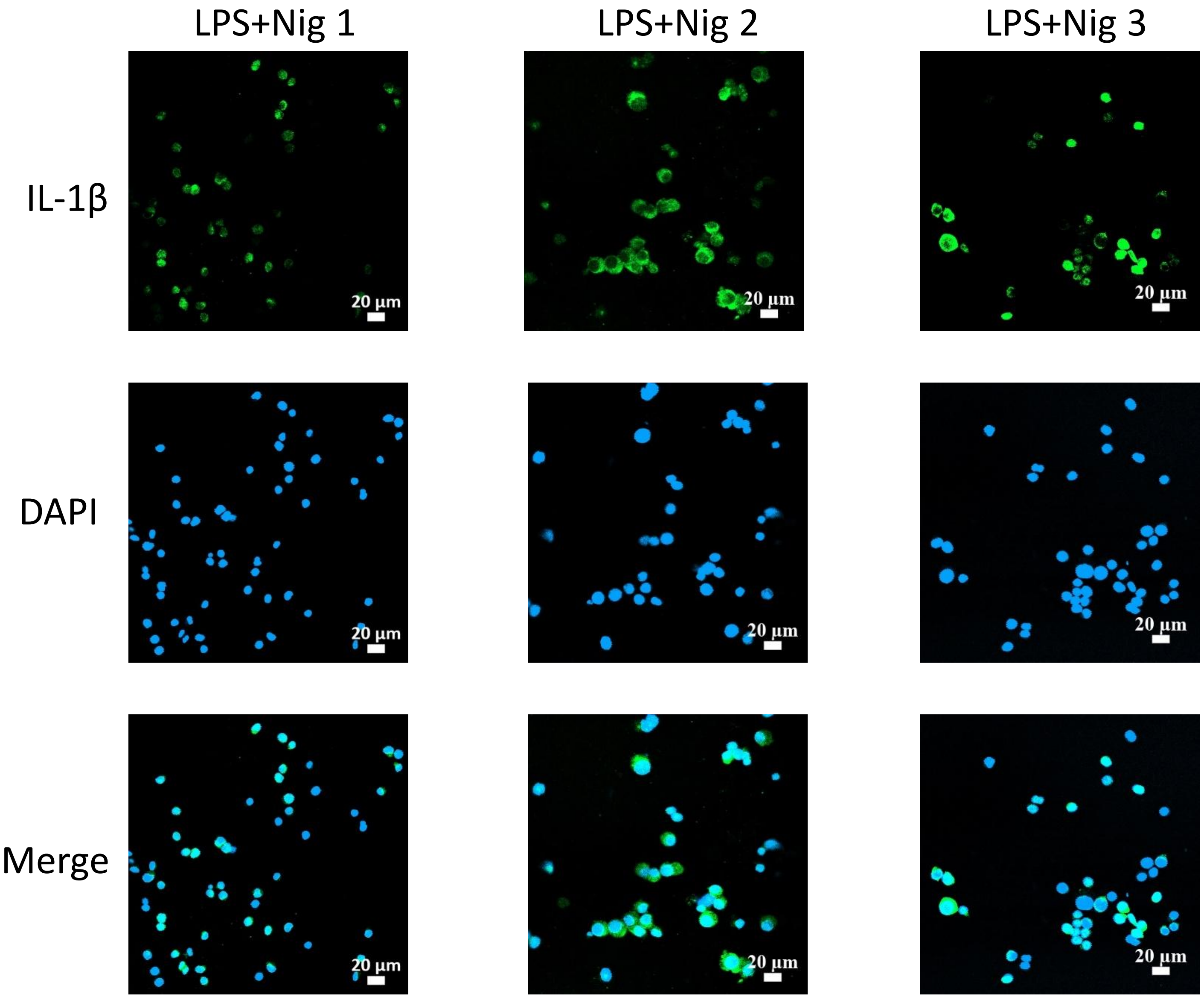

Fig.7B Immunofluorescence analysis（IL-1β）

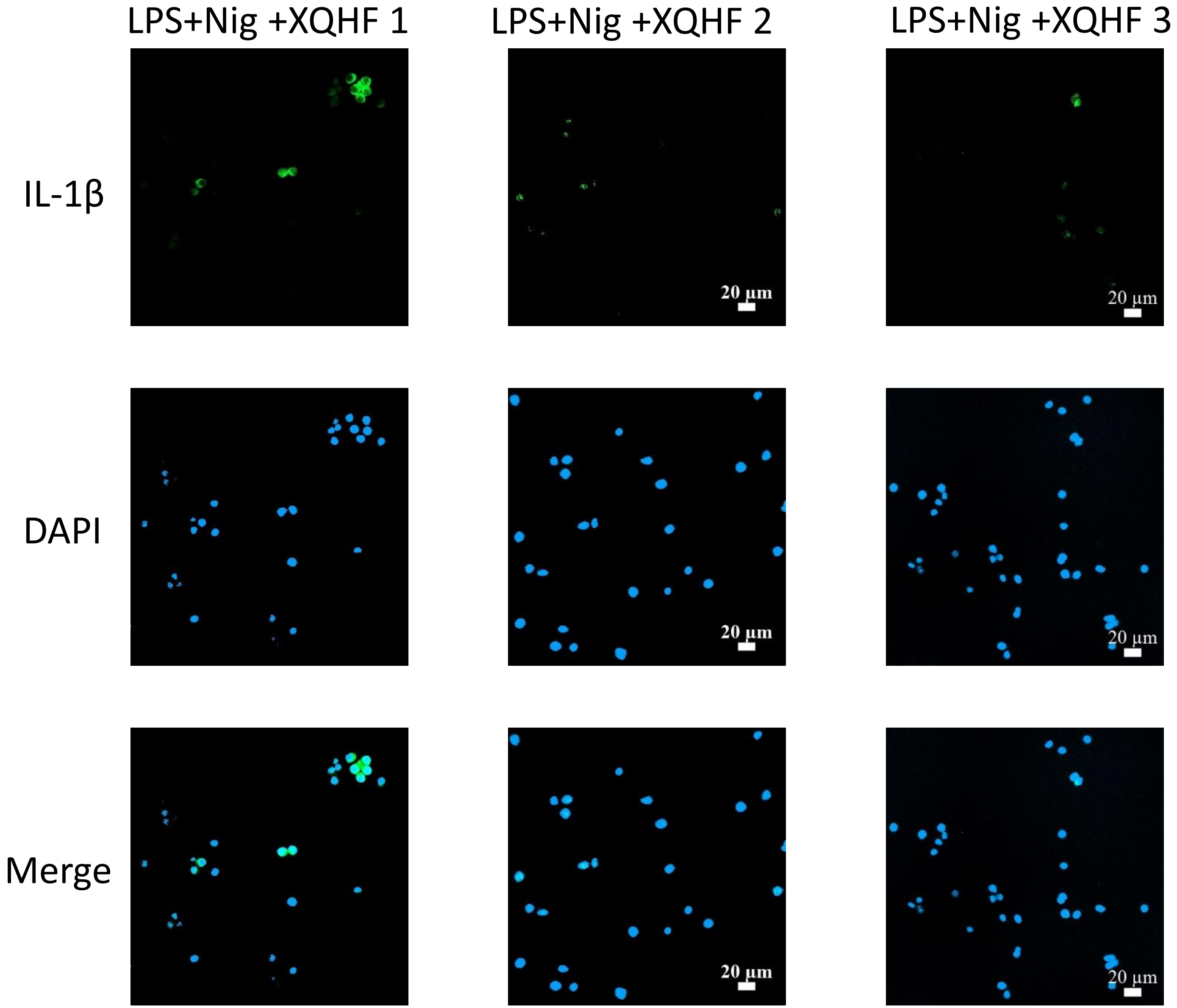

Fig.7B

Immunofluorescence analysis（IL-1β）

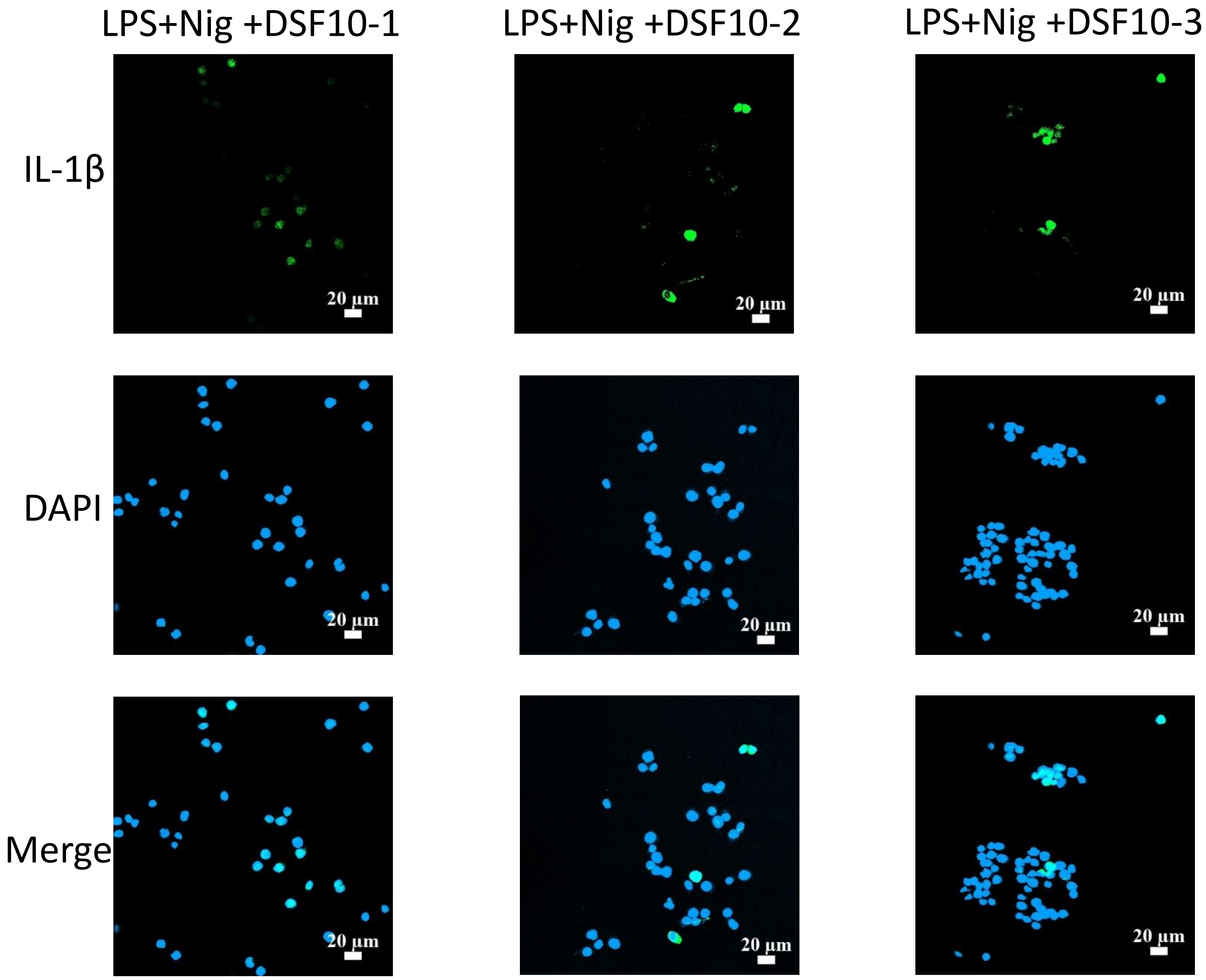

Fig.7C

Immunofluorescence analysis（IL-18）

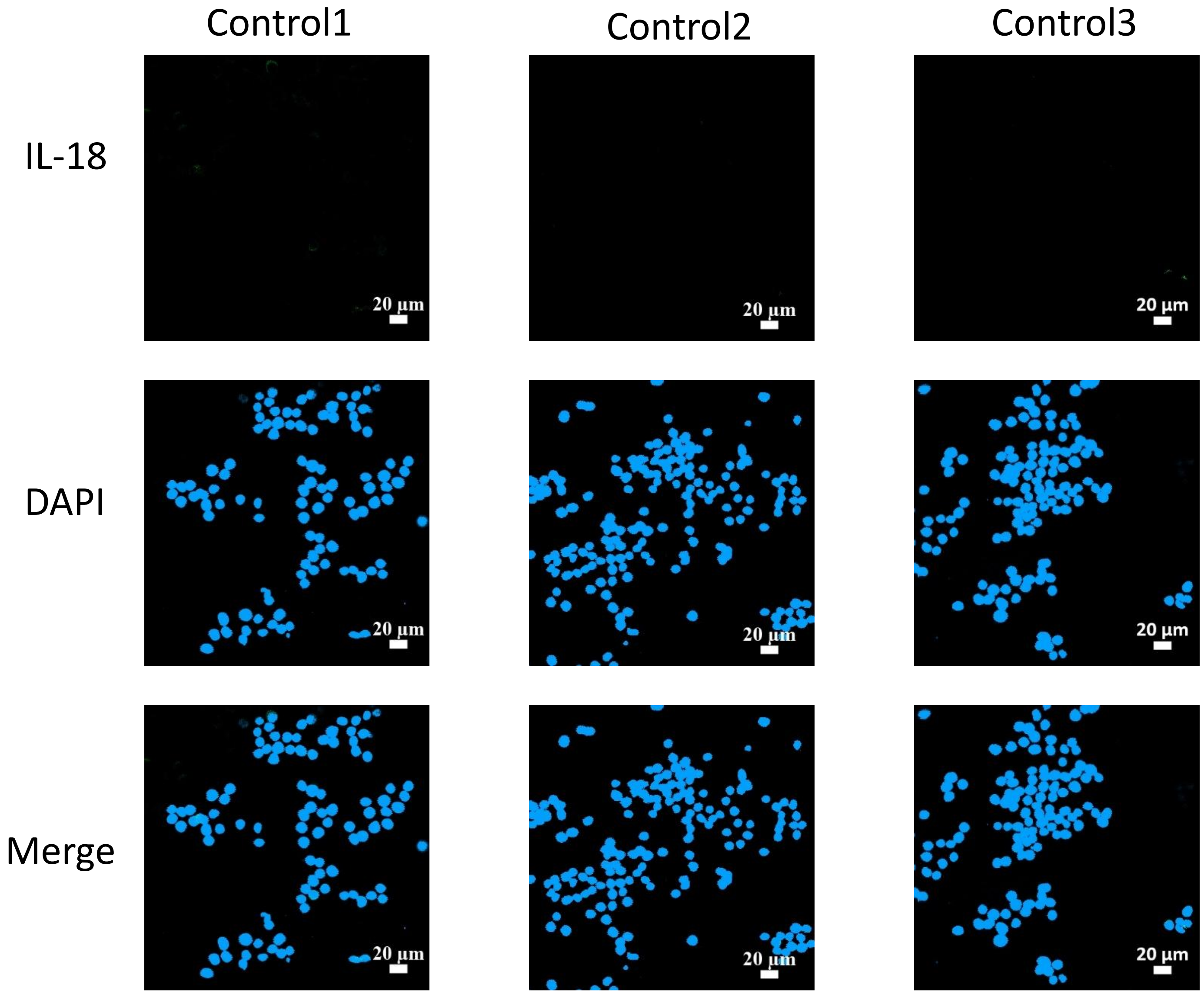

Fig.7C

Immunofluorescence analysis（IL-18）

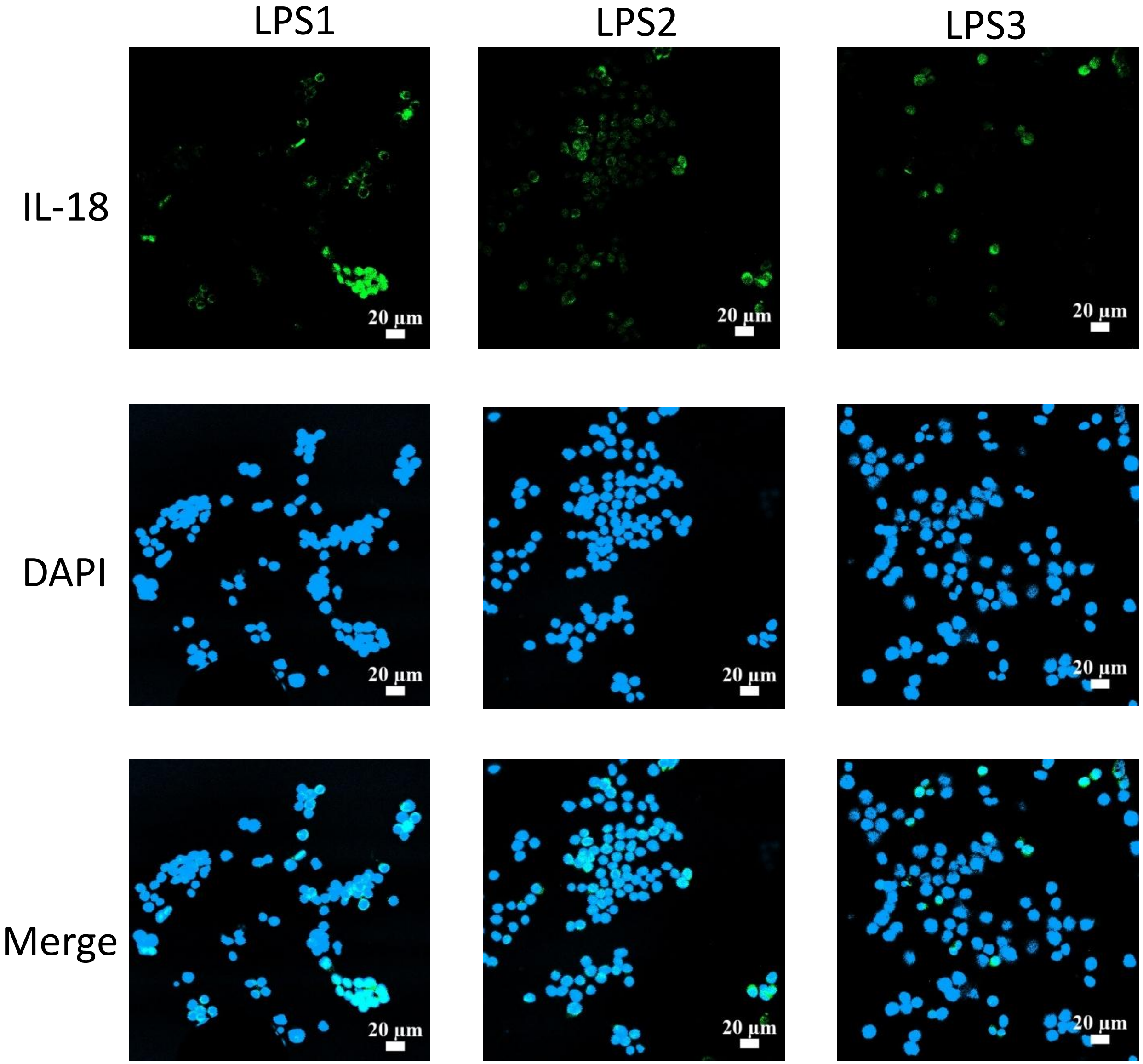

Fig.7C

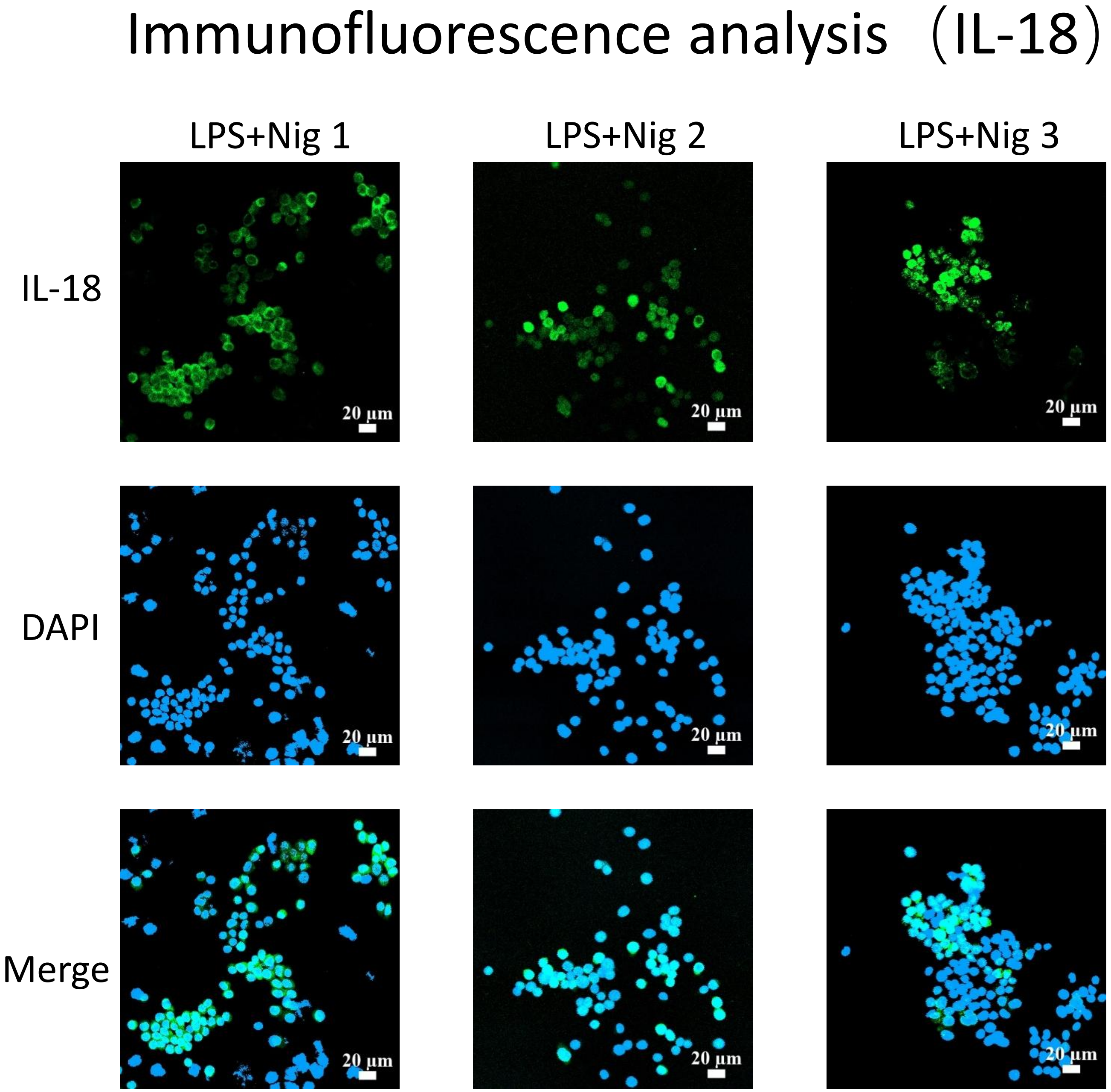
Immunofluorescence analysis（IL-18）
LPS+Nig 1
LPS+Nig 2
LPS+Nig 3
IL-18
DAPI
Merge
20 μm

Fig.7C

Immunofluorescence analysis （IL-18）

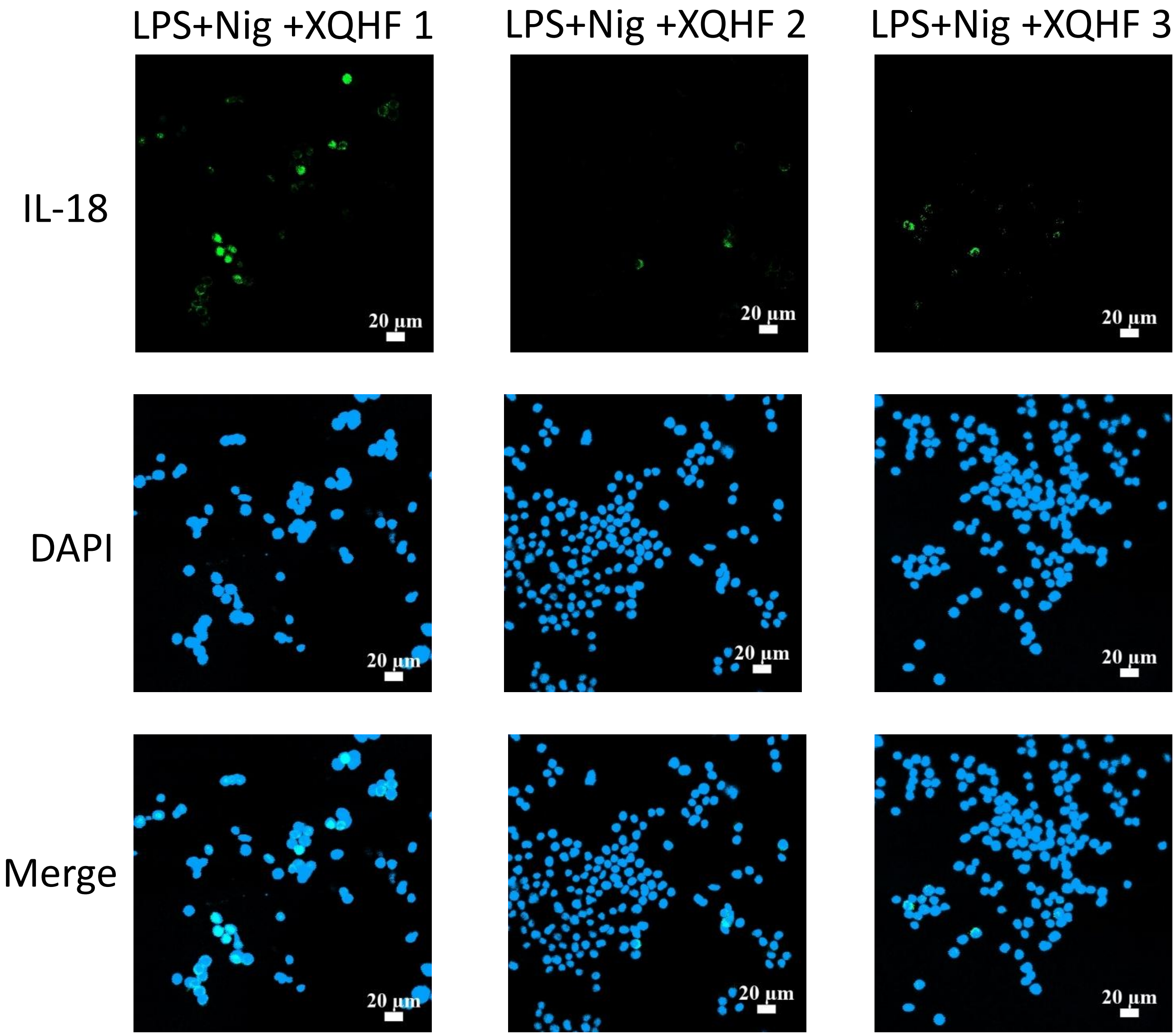

Fig.7C

Immunofluorescence analysis（IL-18）

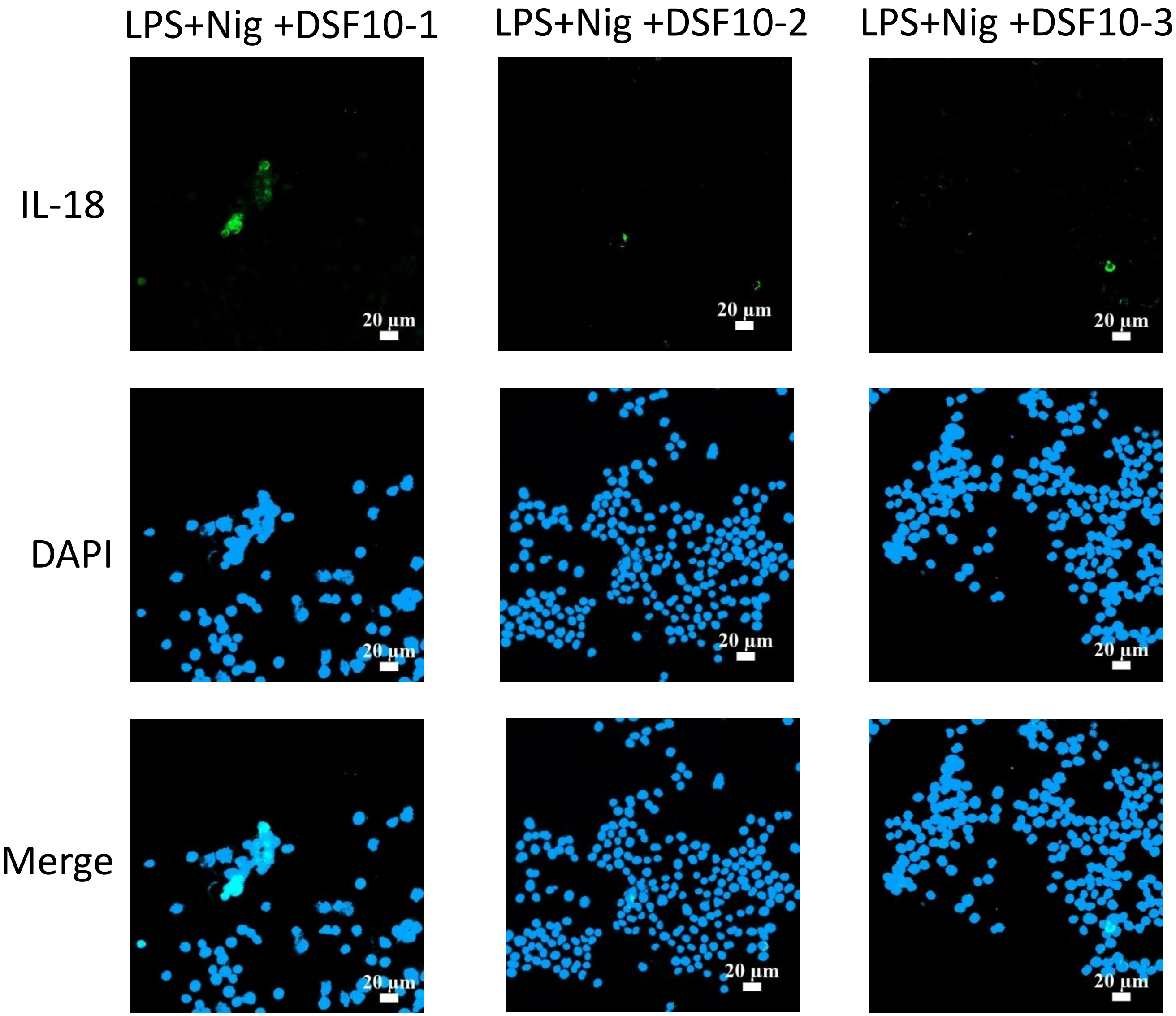

Fig.8 Original blots

NLRP3 （Cell lysate）

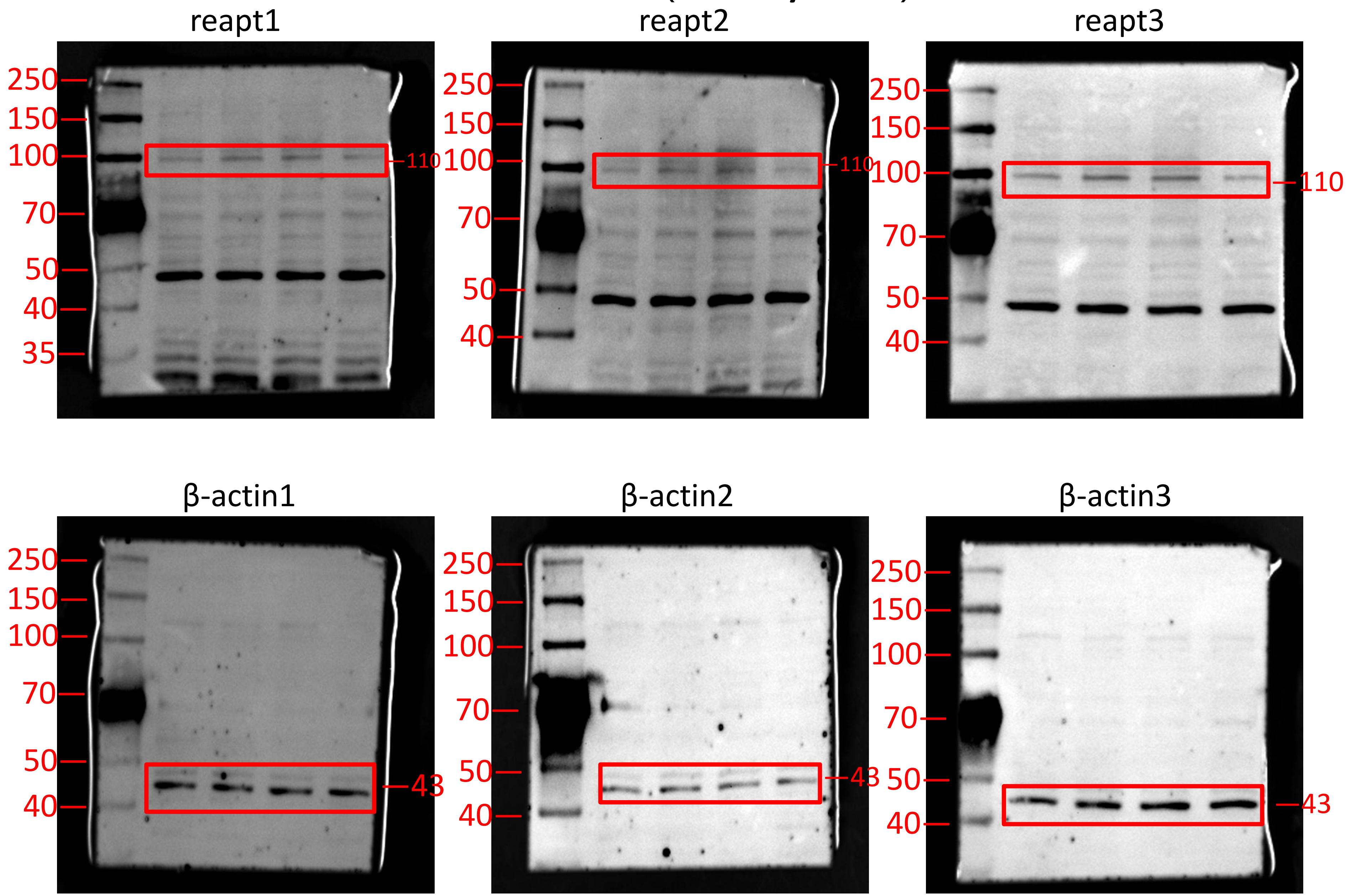

Fig.8 Original blots

Caspase-1 and cleaved-Caspase-1 （Cell lysate）

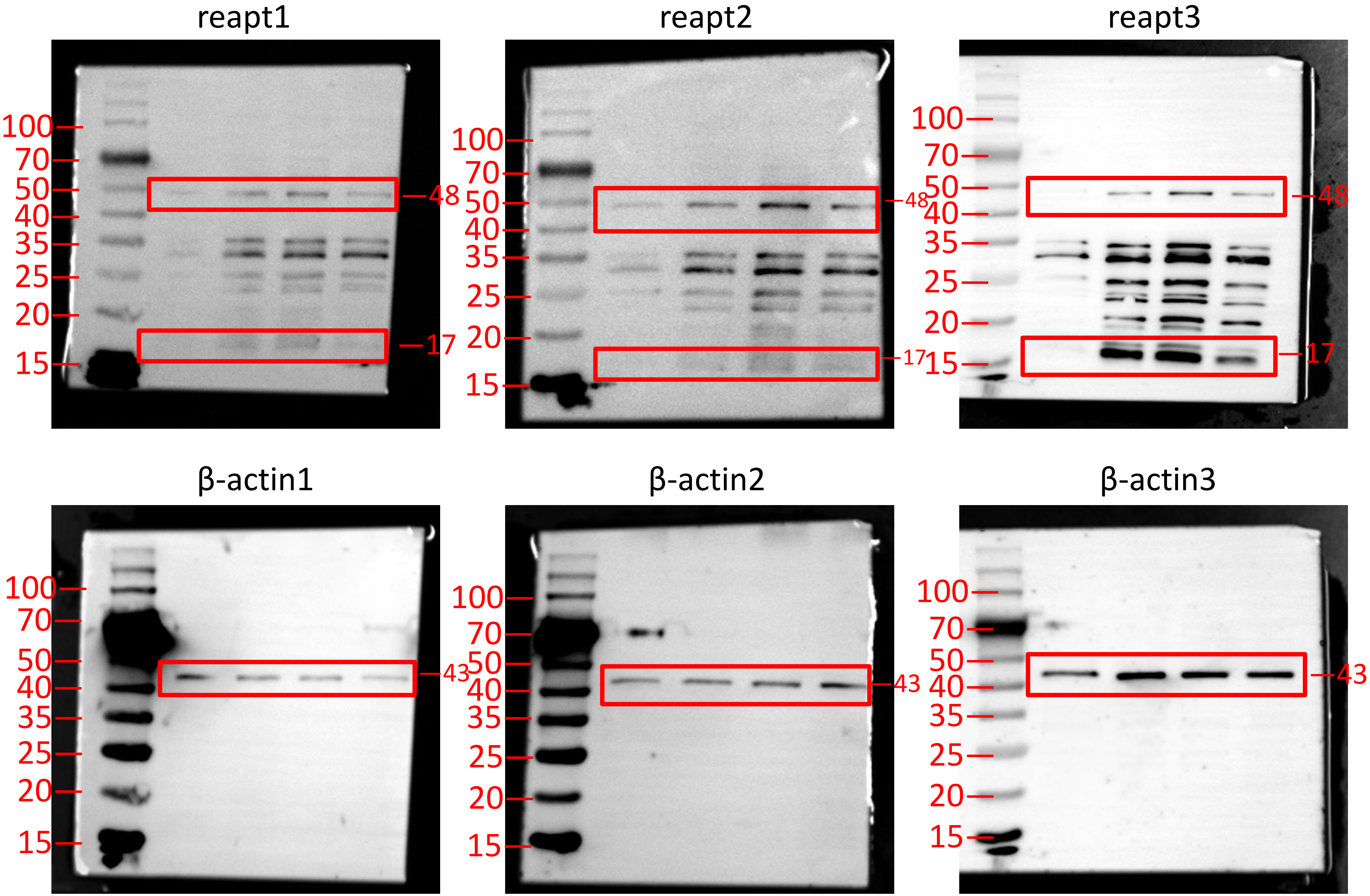

Fig.8 Original blots

GSDMD and GSDMD-N （Cell lysate）

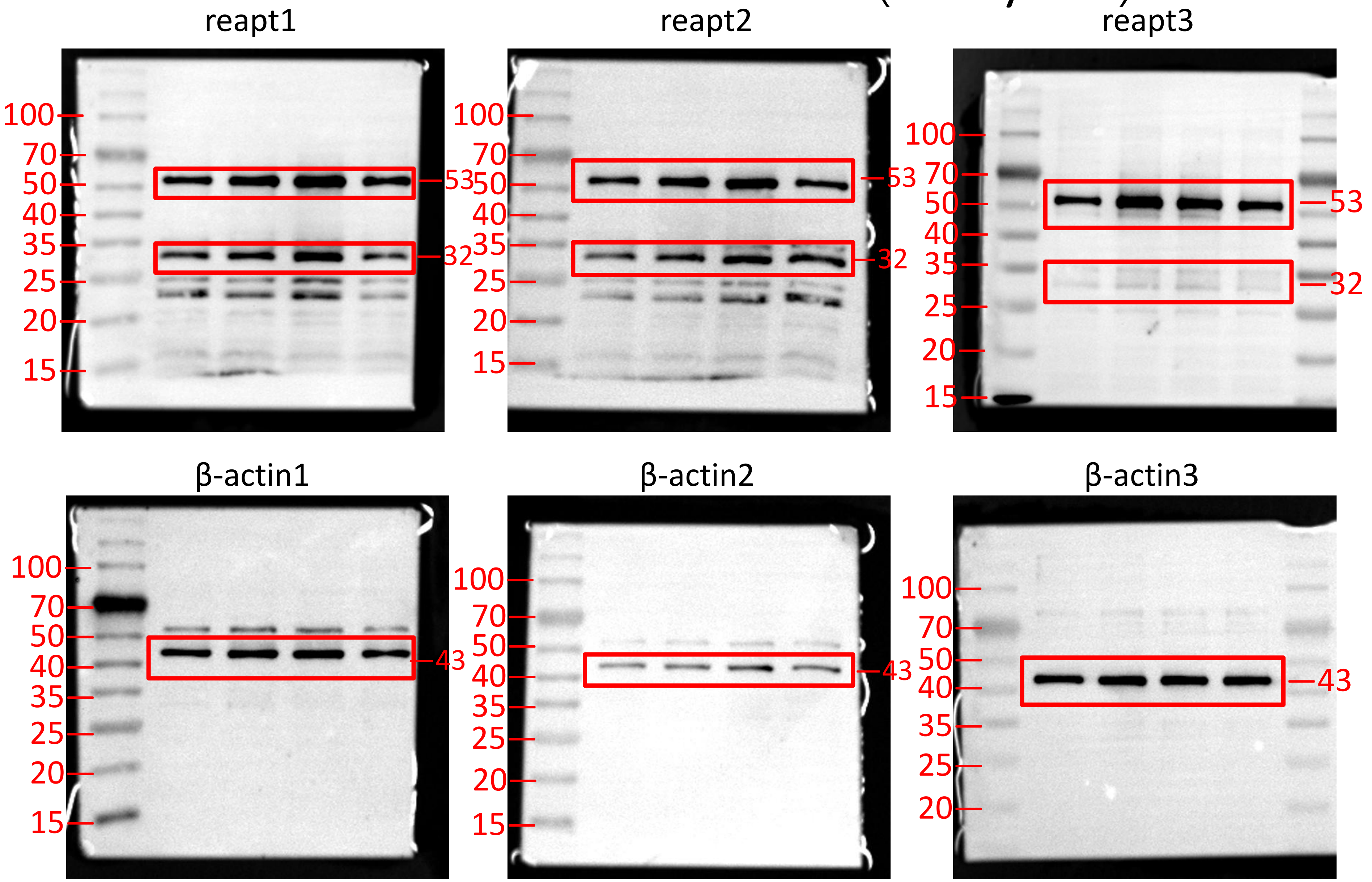

Fig.8 Original blots

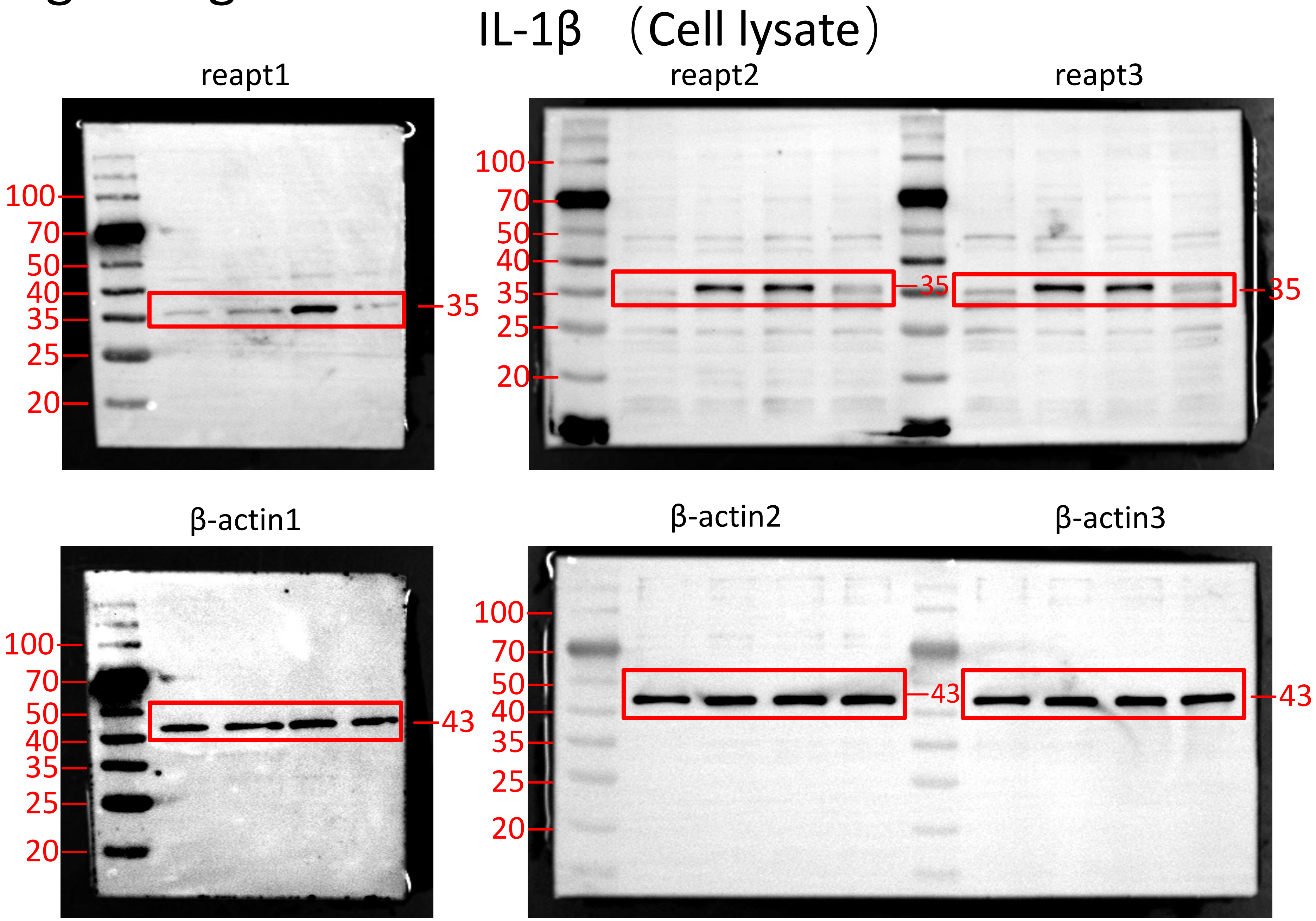

Fig.8 Original blots

NLRP3 （Supernatant）

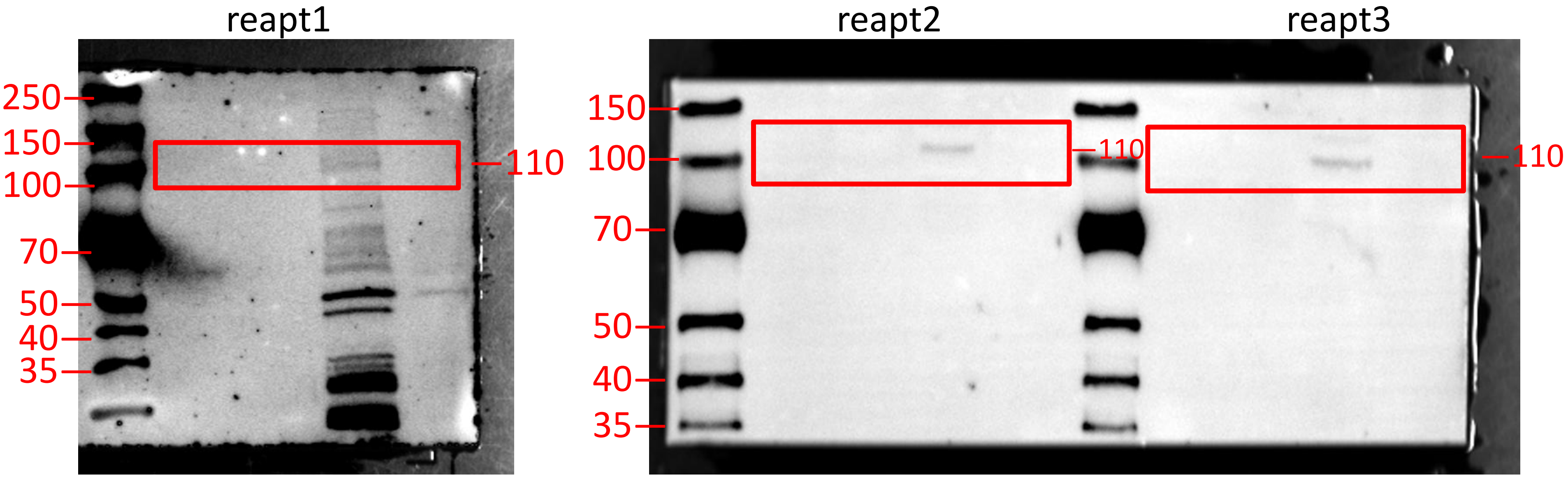

Caspase-1 and cleaved-Caspase-1 （Supernatant）

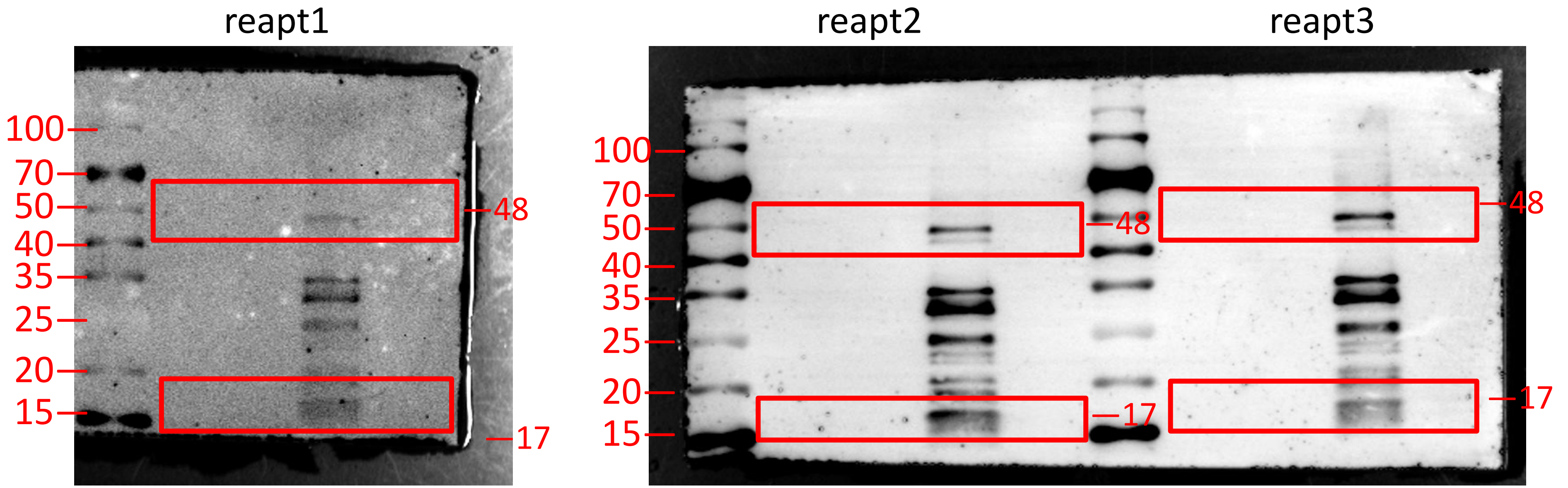

Fig.8 Original blots

GSDMD and GSDMD-N （Supernatant）

reapt1

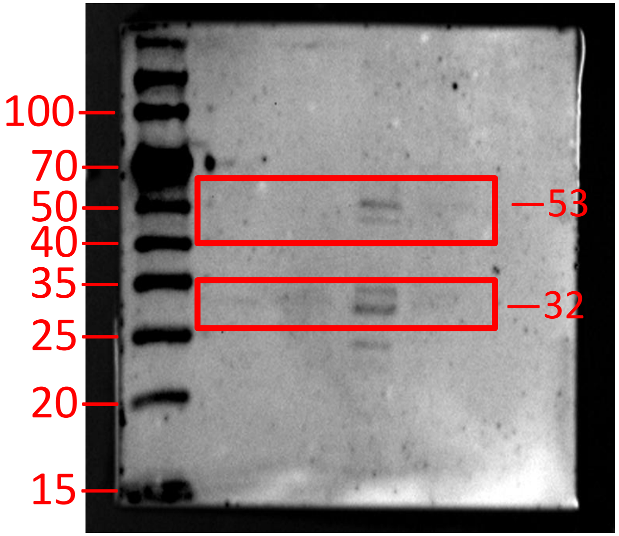

reapt2 reapt3

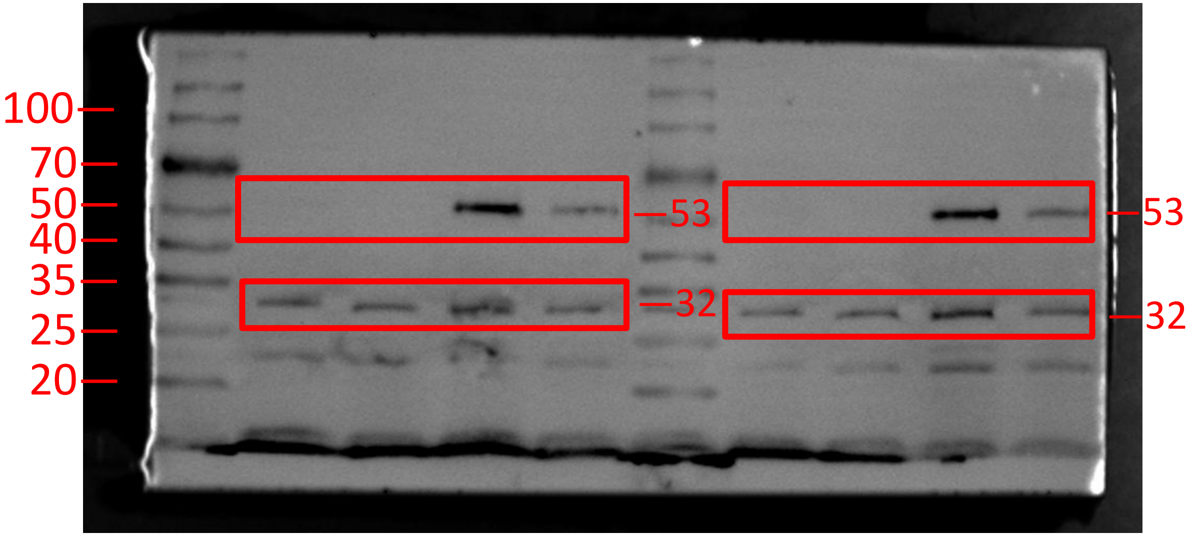

IL-1β （Supernatant）

reapt1

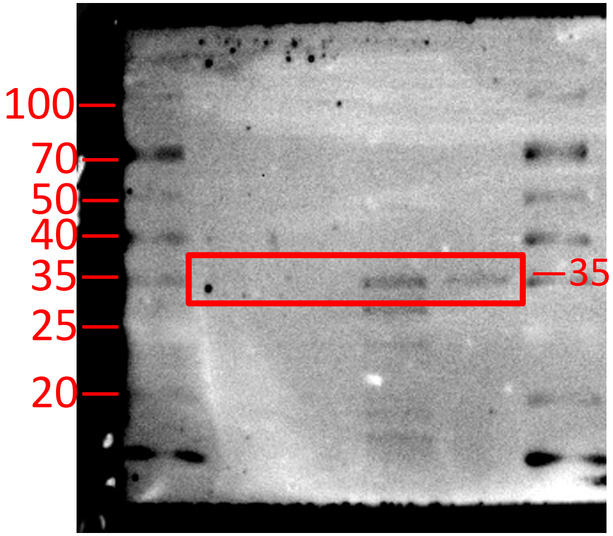

reapt2 reapt3

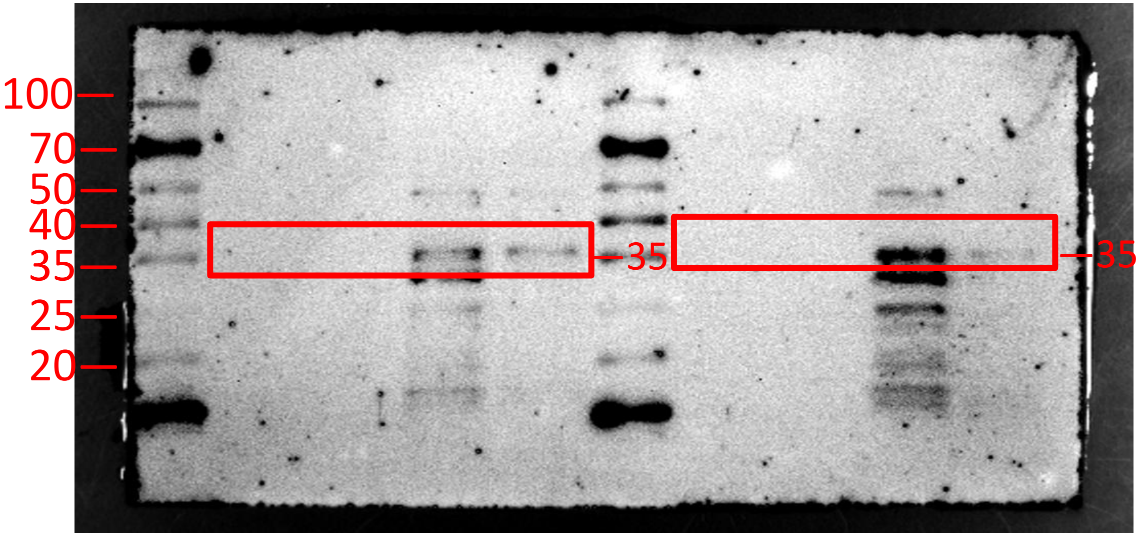

Supplement: Supplementary file 1 [file DataSheet1.pdf]
